# Optimal physical activity type and dosage for improving inhibitory control in children and adolescents: a dose–response network meta-analysis

**DOI:** 10.3389/fpsyg.2026.1702811

**Published:** 2026-03-12

**Authors:** Jirong Wang, Zhanfei Zheng, Zewei Zhou, Mingjia Li

**Affiliations:** 1 School of Police Law Enforcement Abilities Training, People’s Public Security University of China, Beijing, China; 2 Department of Sports Science, Wenzhou Medical University, Wenzhou, Zhejiang, China; 3 College of Science, School of North China University of Technology, Beijing, China; 4 China Basketball College, Beijing Sports University, Beijing, China

**Keywords:** adolescents, children, dose–response, inhibitory control, network meta-analysis, physical activity

## Abstract

**Background:**

Inhibitory control is a key component of executive function, influencing academic achievement, behavior, and long-term outcomes in children and adolescents. Physical activity (PA) has been identified as a potential intervention to improve inhibitory control, but the optimal type and dosage remain uncertain. This study aimed to assess the effects of different PA modalities on inhibitory control and determine the optimal dosage for improving inhibition accuracy in youth.

**Methods:**

A systematic review and network meta-analysis (NMA) were performed on studies published until July 31, 2025. A comprehensive search was conducted across PubMed, Embase, PsycINFO, the Cochrane Central Register of Controlled Trials (CENTRAL), and Web of Science. Thirty-four randomized controlled trials (RCTs) involving 23,209 participants were included. The primary outcomes were inhibition accuracy and inhibition reaction time, with effect sizes calculated as standardized mean differences (SMD) for each outcome. Dose–response analysis was conducted using a Bayesian framework to assess how PA dosage influenced inhibition accuracy and reaction time.

**Results:**

Mixed exercise (ME) significantly improved inhibition accuracy compared to the control group (SMD = 0.52, 95% CrI: 0.21–0.82), with the highest effectiveness according to the surface under the cumulative ranking curve (SUCRA = 0.81). A significant dose–response relationship was observed between PA dosage and inhibition accuracy, with the optimal dosage identified as 1,500 METs-min/week (SMD = 1.08, 95% CrI: 0.29–1.84). No significant dose-effect relationship was found for inhibition reaction time. Snack exercise (SE) exhibited the greatest reduction in inhibition reaction time but had no significant impact on inhibition accuracy.

**Conclusion:**

ME is the most effective PA modality for improving inhibition accuracy, particularly at 1,500 METs-min/week. These findings highlight the importance of PA dosage in optimizing cognitive outcomes in children and adolescents.

**Systematic review registration:**

CRD420251181222.

## 1 Introduction

Inhibitory control is a core component of executive function, referring to the ability to suppress instinctive or prepotent responses in favor of goal-directed behavior (Diamond, 2013). This capacity is especially crucial during childhood and adolescence, as executive functions (including inhibitory control) predict school readiness and academic achievement (Zhou et al., 2025). Strong inhibitory control in youth is associated with better self-regulation and fewer behavioral problems, whereas deficits can manifest as impulsivity and attention difficulties (Mirabella, 2021). Inhibitory control undergoes protracted maturation through childhood and adolescence, and many children and adolescents exhibit underdeveloped self-regulation skills (Constantinidis and Luna, 2019). Given the importance of inhibitory control for learning, social behavior, and long-term life outcomes, there is a growing impetus to identify effective strategies to support its development in typically developing youth (Zeytinoglu et al., 2023). Therefore, it is necessary to explore interventions that can improve inhibitory control in children and adolescents, potentially mitigating developmental gaps and enhancing academic and life success.

Multiple factors influence the development of inhibitory control in young people, including genetic, environmental, and experiential factors. Among these influences, physical activity has emerged as a salient and modifiable factor that can be targeted through intervention (Prentice et al., 2023). Unlike inherent traits or socio-demographic factors, exercise behavior can be readily changed via school or community programs, making it an attractive avenue for enhancing cognitive development. Notably, regular physical activity is known to confer broad benefits for youth—it can improve physical health and fitness while also promoting psychological well-being and social skills (Fu et al., 2025). Importantly, exercise interventions are not only capable of acutely boosting inhibitory control and other executive functions, but they may also instill lifelong habits of regular activity, contributing to healthier lifestyles (Chen et al., 2020). Physical activity has been shown to enhance prefrontal activation, modulate catecholaminergic activity, and improve neural efficiency in frontostriatal circuits, mechanisms that are central to response inhibition and help explain its specific relevance for inhibitory control (Karsai et al., 2023; Wu et al., 2022). These mechanisms are closely linked to response suppression and conflict monitoring, providing a theoretical basis for focusing on inhibition in exercise–cognition research.

Reflecting the potential benefits of exercise, an expanding body of literature has examined the effects of physical activity on inhibitory control in children and adolescents. Numerous studies and meta-analyses indicate that chronic physical activity interventions can produce significant improvements in executive function, including inhibitory control. For example, interventions ranging from traditional aerobic exercise to high-intensity interval training (HIIT) have been reported to enhance youths’ performance on tasks requiring response inhibition (Kao et al., 2023). There is also evidence that the nature of the activity matters; activities that integrate cognitive engagement or complex motor skills (e.g., team sports or coordinative exercises) tend to yield larger cognitive benefits than purely repetitive physical training (Shi and Feng, 2022). A recent network meta-analysis of randomized controlled trials compared the efficacy of various exercise modalities on executive functions in healthy youths, reinforcing that exercise type can modulate cognitive outcomes (Wang et al., 2024). In that analysis, ball games emerged as the most effective activity for improving certain executive function subdomains—notably, they yielded the greatest gains in updating (working memory) accuracy and the fastest inhibition reaction times. Meanwhile, cognitively engaging physical activities (those requiring strategic thinking or coordination) led to the largest improvements in inhibition accuracy, and dance-based exercises were top-ranked for enhancing other executive skills such as cognitive flexibility (shifting). These findings underscore that while physical activity broadly benefits executive function in young people, the magnitude and nature of improvements in inhibitory control may depend on the type of exercise undertaken. However, the effects of exercise may also depend on “dose,” including intensity, frequency, and duration. Evidence suggests that the cognitive benefits of exercise are dose-dependent. Interventions lasting more than 6 weeks, exceeding 20 min per session, and delivered more than twice per week appear particularly effective for enhancing inhibition (Gallardo-Gómez et al., 2022). These dose variations may contribute to heterogeneity in prior findings, highlighting the need to clarify the dose–response relationship to determine both minimal and optimal doses for cognitive benefit (Bull et al., 2020). This aspect remains insufficiently examined in youth populations. In addition, inhibition represents a theoretically meaningful focus because it shows substantial developmental plasticity and is strongly linked to academic readiness and behavioral regulation. Compared with working memory, inhibition may be more sensitive to physical activity-induced changes, offering a clearer target for identifying exercise-related cognitive enhancement.

Given the above background, the present study was designed to address these knowledge gaps by using a model-based Bayesian framework for dose–response network meta-analysis (NMA). Network meta-analysis offers the advantage of comparing multiple intervention types within a single analytical framework, even when direct head-to-head trials are lacking (Leucht et al., 2016). This approach allows for a comprehensive ranking of the effectiveness of different exercise modalities, providing a broader evidence base for recommendations. By extending NMA to incorporate dose–response modeling, this study simultaneously evaluates the relative efficacy of different exercise types and quantifies how exercise dose influences improvements in inhibitory control among healthy children and adolescents. The aim of this research is twofold: first, to determine which types of physical activity are most effective for enhancing inhibitory control in youth; and second, to elucidate the dose–response relationship between exercise and inhibitory control outcomes. This work is expected to fill an important gap in current knowledge by integrating both intervention modality and dosage into a unified analysis. Clarifying these factors has significant practical implications, as it can guide educators, coaches, and public health practitioners in designing evidence-based physical activity programs to optimally support cognitive development. Ultimately, by identifying effective exercise strategies and their required doses for cognitive benefit, the findings of this study could help maximize the neurocognitive benefits of physical activity in children and adolescents and inform targeted interventions to improve inhibitory control at the population level.

## 2 Methods

This systematic review and network meta-analysis was conducted and reported in accordance with the Preferred Reporting Items for Systematic Reviews and Meta-Analyses (PRISMA) 2020 statement and its extension for network meta-analyses (Hutton et al., 2015; Page et al., 2021). The review protocol was prospectively registered in the PROSPERO database. Given that the analysis involved only previously published data without direct patient involvement, ethical approval and informed consent were not required.

### 2.1 Data sources and search strategy

A comprehensive and systematic literature search was performed across PubMed, Embase, PsycINFO, the Cochrane Central Register of Controlled Trials (CENTRAL), and Web of Science from database inception to July 31, 2025. The key search terms included combinations of “children,” “adolescents,” “exercise,” and “inhibitory control.” The detailed search strategies for each database are provided in Supplementary File 2. Additionally, reference lists of included studies and recent systematic reviews published within the past 5 years were carefully screened to identify potentially eligible articles. Two authors independently conducted title, abstract, and full-text screening, with any disagreements resolved through discussion or adjudication by a third reviewer.

### 2.2 Eligibility criteria

Studies meeting the following criteria were eligible for inclusion: (1) Participants were healthy children aged 6–12 years or adolescents aged 13–18 years; (2) interventions involved structured exercise programs, categorized into five distinct exercise types (with explicit inclusion of “exercise snacks” as a separate modality, defined as intermittent bouts of short-duration exercise) detailed in Supplementary File 3; (3) control groups included no intervention or health education courses without physical activity; (4) exercise interventions lasted at least 4 weeks, allowing evaluation of chronic effects on inhibitory control; (5) reported outcomes included measures of inhibitory control, specifically accuracy and reaction time; and (6) randomized controlled trial (RCT) design.

Exclusion criteria were: (1) inclusion of participants with cognitive impairments, attention deficit hyperactivity disorder (ADHD), or professional athletes; (2) interventions combining cognitive training with physical activity, aiming to isolate the specific effects of physical exercise on inhibitory control; (3) multicomponent lifestyle interventions (e.g., combined nutrition and exercise); (4) studies focusing on acute rather than chronic exercise effects; (5) studies that failed to specify exercise type or quantify exercise dose clearly; and (6) unavailable data on means and standard deviations after attempting to contact authors at least four times within 6 weeks.

### 2.3 Data extraction

Two independent reviewers extracted relevant data from the included studies, including basic study information (authors, title, publication year), participant characteristics (sample size, age, gender), intervention details (type, intensity, frequency, duration of exercise), and outcome measures related to inhibitory control. The main characteristics of included studies are summarized in Table 1.

**Table 1:** Characteristics of studies and subjects included in the review.

Study	Subjects (intervention/control)	Sex (boy/girl) (intervention/control)	Mean age (intervention/control)	BMI (intervention/control)	Intervention detail		METs	Session duration	Training frequency	Duration	Adherence	Inhibition accuracy	Inhibition reaction time
					Intervention group	Control group							
Alesi et al. (2016)	44 (24/20)	24/0 vs. 20/0	8.8 ± 1.1 vs. 9.3 ± 0.9	19.2 ± 4.5 vs. 21.5 ± 4.7	ME: soccer training, including individual skills, technique or/and one-on-one situations, opposed games involving three-on-three and five-on-five	Usually activities	5.4	60 min	2 × per week	6 months	N/A	Postintervention, Go/No-Go task	N/A
Barboza et al. (2021)	61 (34/27)	13/21 vs. 14/13	7.7 ± 0.5 vs. 8.0 ± 0.7	16.0 ± 2.1 vs. 17.0 ± 2.9	ME: spell out letters and numbers physically or play board games and jigsaw puzzles; perform dance routines to music.	Not receive any intervention	4.7	15 min	3 × per week	36 weeks	N/A	Postintervention, Go/No-Go task	Postintervention, Go/No-Go task
Beck et al. (2016)	102 (55/57)	30/25 vs. 28/29	7.5 ± 0.02 vs. 7.5 ± 0.02	16.5 ± 0.3 vs. 16.5 ± 0.3	ME: includes coordination, aerobic and static movements for jumping, climbing, hopping, throwing, and balancing on one foot.	Usually activities	5	60 min	5 × per week	6 weeks	91.30%	Postintervention, Flanker task	Postintervention, Flanker task
Beckmann et al. (2022)	481 (257/224)	121/136 vs. 126/98	8.8 ± 2.9 vs. 8.1 ± 1.3	N/A	DC: moving to music esson	Usually activities	3.6	45 min	2 × per week	12 weeks	N/A	Postintervention, Flanker task	Postintervention, Flanker task
Cho et al. (2017)	30 (15/15)	9/6 vs. 9/6	11.2 ± 0.8 vs. 11.3 ± 0.7	21.6 ± 4.0 vs. 20.8 ± 1.9	ME: basic movements, Poomsae, Taekwondo kicks and steps, practice mitt kicking, and Taekwon gymnastics	Usually activities	6.0	50 min	5 × per week	16 weeks	N/A	Postintervention, Stroop Color-Word Task	N/A
Contreras-Osorio et al. (2022)	90 (30/30/30)	16/14 vs. 16/14 vs. 15/15	11.4 ± 0.6 vs. 11.4 ± 0.9 vs.11.5 ± 0.6	19.3 ± 2.4 vs. 19.2 ± 3.1 vs. 19.3 ± 3.7	(a) ME: Individual improvement tasks, one-phase improvement tasks, combination tasks, and real play. (b) AE: Athletic abilities with a technical component, lifts and repetitions through the set, introduction to fartlek, and continuous running.	Usually activities	(a) SG: 6.2 (b) AE: 7.0	40 min	2 × per week	12 weeks	> 80%	Postintervention, Interference	N/A
Drollette et al. (2018)	308 (139/169)	68/71 vs. 83/86	8.72 ± 0.05 vs. 8.75 ± 0.04	N/A	ME: aerobic exercise, resistance training, motor skill acquisition.	Usually activities	3.2	70 min	5 × per week	9 months	85.40%	Postintervention, Flanker task	Postintervention, Flanker task
Egger et al. (2019)	142 (49/47/46)	21/28 vs. 21/26 vs. 22/24	7.9 ± 0.4 vs. 7.9 ± 0.4 vs. 7.8 ± 0.4	16.51 ± 2.91 vs. 16.21 ± 2.22 vs. 16.21 ± 2.36	(a) AE: Mimic the teacher in aerobic movements such as horse racing and jumping. (b) SG: Standing in a circle and playing the game “Horserace.”	Cognitive activities without PA	(a) AE: 4.6 (b) SG: 5.9	10 min	2 × per day, 5 × per week	20 weeks	N/A	Postintervention, Flanker task	Postintervention, Flanker task
Gentile et al. (2020)	411 (211/200)	197 vs. 214	9.6 ± 1.8 vs. 9.6 ± 1.8	N/A	ME: Movements are completed through instructions and include a variety of elements such as coordination, aerobic and physical play.	Standard PE lessons	6.1	25 min	5 × per week	14 weeks	N/A	Postintervention, Stroop test	N/A
Hillman et al. (2014)	221 (109/112)	56/53 vs. 63/49	8.8 ± 0.2 vs. 8.8 ± 0.2	19.1 ± 1.19 vs. 18.9 ± 1.1.8	ME: Aerobic exercise, resistance training, motor skill acquisition.	Usually activities	3.2	70 min	5 × per week	9 months	80.60%	Postintervention, Flanker task	Postintervention, Flanker task
Krafft et al. (2014)	43 (24/19)	7/17 vs. 8/11	9.7 ± 0.8 vs. 9.9 ± 0.9	1.91 ± 0.41 vs. 1.93 ± 0.57(BMI z-score)	AE: Aerobic activities (e.g., tag and jump rope)	Instructor-led sedentary activities	6.0	40 min	5 × per week	8 months	58.00%	Postintervention, Flanker task	Postintervention, Flanker task
Kvalø et al. (2017)	449 (227/222)	230 vs. 219	9.6 ± 0.7 vs. 9.3 ± 0.6	N/A	ME: Running to answer questions, Capture the Flag, Tag, Jump Rope, Strength Training.	Regular PE lessons	6.2	65 min	2 × per week	40 weeks	N/A	Postintervention, Stroop test	N/A
Leahy et al. (2020)	62 (33/29)	18/15 vs. 14/15	16.2 ± 0.4 vs. 16.2 ± 0.4	N/A	SE: Combination of aerobic exercise and resistance exercises (e.g., star jumps, squat jumps)	Usually activities	6.4	12–20 min	3 × per week	14 weeks	N/A	Postintervention, Flanker task	Postintervention, Flanker task
Logan et al. (2021)	103 (56/47)	27/29 vs. 18/29	8.6 ± 0.6 vs. 8.8 ± 0.7	N/A	ME: Fitness stations, dance and motor skill development, and small-sided games such as 3v3 soccer	Regular after-school routine	5.7	24 min	5 × per week	9 months	79.97%	Postintervention, Flanker task	Postintervention, Flanker task
Lubans et al. (2020)	670 (337/333)	168/169 vs. 203/130	16.0 ± 0.4 vs. 16.0 ± 0.5	N/A	SE: Combination of aerobic exercise and self-weighted resistance exercises (e.g., round-trip running, open-close jumps, push-ups)	Usually activities	7.4	8–20 min	2 × per week	6 months	N/A	Postintervention, Flanker task	Postintervention, Flanker task
Ludyga et al. (2019a)	45 (15/15/15)	25 vs. 20	9.1 ± 0.6 vs. 9.6 ± 0.8 vs. 9.3 ± 0.6	16.2 ± 4.4 vs. 17.5 ± 3.2 vs. 16.9 ± 1.5	(a) AE: Running-based games at moderate-to-vigorous intensity; (b) SG: Playful exercise games in coordination such as balance, bilateral coordination, and spatial orientation.	Assisted homework sessions	(a) AE: 3.5 (b) CE: 2.3	45 min	3 × per week	10 weeks	>90%	Postintervention, Flanker task	Postintervention, Flanker task
Ludyga et al. (2019b)	36 (20/16)	23 vs. 13	12.5 ± 0.9 vs. 12.4 ± 0.8	18.2 ± 2.2 vs. 19.7 ± 3.6	ME: Combination of aerobic and coordinated exercises, e.g., relay games, ball games.	Talks with classmates	3.2	20 min	5 × per week	8 weeks	N/A	Postintervention, Stroop test	Postintervention, Stroop test
Ludyga et al. (2021)	42 (22/20)	13/9 vs. 10/10	10.3 ± 1.2 vs. 10.7 ± 1.5	17.7 ± 2.3 vs. 18.2 ± 3.5	ME: Physical fitness training, technique learning, and free fighting.	Usually activities	6.0	60 min	2 × per week	3 months	89.70%	Postintervention, Go/No-Go task	N/A
Mavilidi et al. (2020)	58 (29/29)	14/15 vs. 20/9	8.7 ± 0.5 vs. 9.2 ± 0.6	N/A	SE: Combat (straight and cross-over punches, squats), fitness (skipping, jumping jacks, jogging on the spot), and cardio (lunges, skater jumps, push ups).	Mathematical activities	4	5 min	3 × per week	4 weeks	N/A	Postintervention, Flanker task	Postintervention, Flanker task
Meijer et al. (2020)	621 (206/235/415)	102/104 vs. 109/126 vs. 209/206	9.3 ± 0.7 vs. 9.0 ± 0.6 vs. 9.2 ± 0.7	16.8 ± 2.1 vs. 16.8 ± 2.3 vs. 16.5 ± 2.3	(a) AE: Circuit training, relay games, playing tag, and individual activities like running. (b) SG: Team games or exercises include dodgeball, basketball, or soccer.	Regular PE lessons	(a) AE: 6.5 (b) SG: 5.8	20 min	(a) AE: 4 × per week (b) SG: 4 × per week (c) CON: 2 × per week	14 weeks	N/A	N/A	Postintervention, Stop Signal Task
Mora-Gonzalez et al. (2023)	67 (35/32)	24/11 vs. 17/15	10.0 ± 1.1 vs. 10.1 ± 1.1	27.1 ± 4.1 vs. 26.1 ± 2.9	ME: (a) 4–5 moderate to high intensity cardio games (60 min); (b) Resistance training for muscle and bone strengthening (20 min).	Usually activities	5.3	80 min	3 × per week	20 weeks	70	Postintervention, Flanker task	Postintervention, Flanker task
Robinson et al. (2022)	73 (29/23)	12/17 vs. 15/8	15.9 ± 0.4 vs. 15.8 ± 0.4	N/A	SE: Resistance training in Tabata mode, 20 s of work/10 s of rest × 8 sets, total workout time 4 min	Sedentary control	3.5	4 min	3 × per week	4 weeks	N/A	Postintervention, Flanker task	N/A
Roh et al. (2018)	30 (15/15)	9/6 vs. 9/6	11.5 ± 0.6 vs. 11.4 ± 0.6	20.2 ± 4.3 vs. 20.5 ± 3.3	ME: Taekwondo motions, Poomsae, kicking sessions, Taekwon gymnastics.	Usually activities	4.5	50 min	1 × per week	16 weeks	N/A	Postintervention, Stroop color and word test	N/A
Schmidt et al. (2015)	181 (69/57/55)	26/43 vs. 28/29 vs. 28/27	11.3 ± 0.6 vs. 11.3 ± 0.6 vs. 11.4 ± 0.6	18.2 ± 2.8 vs. 17.4 ± 2.5 vs. 17.6 ± 2.6	(a) SG: Team games including tag, floorball and basketball. (b) AE: Different group-oriented and playful forms of aerobic exercises.	Regular PE lessons	(a) SG: 4.5; (b) AE: 5.0.	45 min	2 × per week	6 weeks	N/A	N/A	Postintervention, Flanker task
St Laurent et al. (2019)	53 (27/26)	18/9 vs. 11/15	8.8 ± 0.1 vs. 9.4 ± 0.1	N/A	SG: 25–40% muscular fitness and 60–75% cardiorespiratory bingo games.	Standard practice	3.4	13 min	5 × per week	3 months	30.10%	Postintervention, Flanker task	N/A
Takehara et al. (2021)	1878 (941/937)	481/460 vs. 488/449	9.7 ± 0.4 vs. 9.7 ± 0.4	N/A	SE: Exercise program combined with music, included jumps, squats, and various steps.	Regular PE lessons	6.1	3–5 min	2 × per week	10 weeks	N/A	N/A	Postintervention, Flanker task
Tocci et al. (2022)	95 (46/49)	23/23 vs. 24/25	7.7 ± 1.2 vs. 7.8 ± 1.4	17.8 ± 2.9 vs. 18.0 ± 3.0	ME: Spell out letters and numbers physically or play board games and jigsaw puzzles; perform dance routines to music.	Not receive any intervention	4.7	60 min	1 × per week	24 weeks	N/A	Postintervention, Random Number Generation	N/A
Torbeyns et al. (2017)	44 (21/23)	8/13 vs. 13/10	14.3 ± 0.6	19.7 ± 3.5 vs. 20.1 ± 3.7	AE: cycling on a height-adjustable bike table (LifeSpan C3-DT5 Bike Table).	Not receive any intervention	6.3	40 min	5 × per week	15 weeks	66.70%	Postintervention, Stroop Color-Word Task	Postintervention, Stroop Color-Word Task
van den Berg et al. (2019)	512 (263/249)	142/121 vs. 132/117	10.8 ± 0.6 vs. 10.9 ± 0.7	17.2 ± 2.3 vs. 17.3 ± 2.3	DC: consists of three “Just Dance” videos in which children are asked to imitate dancing.	Educational lessons	2.5	10 min	5 × per week	9 weeks	89.00%	Postintervention, Stroop Color-Word Task	Postintervention, Stroop Color-Word Task
Vazou et al. (2020)	39 (22/17)	11/11 vs. 10/7	7.6 ± 1.5 vs. 7.8 ± 1.6	17.1 ± 3.2 vs. 16.4 ± 2.6	DC: rhythmically jumping on elastic bands to the beat of the song.	Regular PE lessons	3.5	30 min	2 × per week	7 weeks	86.36%	Postintervention, Flanker task	Postintervention, Flanker task
Veldman et al. (2020)	60 (30/30)	16/14 vs. 14/16	7.6 ± 1.8 vs. 7.8 ± 1.9	N/A	SG: consists of team game sports that rotate between football, netball, basketball and touch/oztag, or themed sessions based on popular TV shows such as The Amazing Race and Survivor.	Usually activities	6.0	75 min	3 × per week	24 weeks	N/A	Postintervention, Go/No-Go task	N/A
Wassenaar et al. (2021)	16,017 (7,860/8157)	3394/4466 vs. 3,662/4495	12.5 ± 0.3 vs. 12.5 ± 0.3	N/A	SE: aerobics consist of squats and lunges, and sprinting on the spot.	Regular PE lessons	6.3	10 min	2 × per week	10 months	N/A	Postintervention, Flanker task	Postintervention, Flanker task
Zhang et al. (2023)	100 (49/50)	49 vs. 50	6.2 ± 0.3 vs. 6.1 ± 0.3	16.2 ± 1.8 vs. 17.2 ± 2.7	AE: running, stretching, jumping rope, sport games.	Usually activities	3.2	30 min	4 × per week	11 weeks	N/A	Postintervention, Go/No-Go task	N/A
Zinelabidine et al. (2022)	41 (19/22)	10/9 vs. 12/10	10.3 ± 0.6 vs. 10.0 ± 0.4	17.4 ± 3.1 vs. 19.2 ± 5.5	DC: continuous aerobic dance exercises.	Regular PE lessons	2.7	30 min	2 × per week	8 weeks	N/A	Postintervention, Stroop test	N/A

N/A, none available; AE, aerobic exercise; CON, control group; DC, dance; ME, mixed exercise; SE, snack exercise; SG, sports game.

To calculate effect sizes, mean changes (post-intervention minus baseline scores), standard deviations (SD), and sample sizes were extracted. When mean changes and SDs were not reported explicitly, these values were estimated following recommended methods described in the Cochrane Handbook (Akl et al., 2019). If data remained unavailable, authors were contacted as described above.

### 2.4 Risk of bias and quality of evidence

The risk of bias for each included study was assessed independently by two reviewers using the Cochrane Risk of Bias 2.0 tool (Sterne et al., 2019), covering domains of random sequence generation, deviations from intended interventions, incomplete outcome data, measurement bias, and selective outcome reporting. Disagreements were resolved through discussion or consultation with a third reviewer.

The quality and certainty of evidence for each outcome was further evaluated using the Confidence in Network Meta-Analysis (CINeMA) online application, assessing six domains: within-study bias, reporting bias, indirectness, imprecision, heterogeneity, and inconsistency. Each outcome was categorized into four confidence levels: high, moderate, low, or very low (Nikolakopoulou et al., 2020).

### 2.5 Data coding and management

Interventions were coded and analyzed at three hierarchical levels. At the primary level, interventions were categorized as physical activity (PA) versus control (CON). At the secondary level, PA interventions were further classified into specific exercise modalities: aerobic exercise (AE), dance (DC), mixed exercise (ME), exercise snacks (SE), or sport games (SG). To ensure consistent classification, operational criteria were applied. AE referred to continuous rhythmic movement at moderate-to-vigorous intensity; DC involved choreographed rhythmic movement; ME included programs combining aerobic, strength, or coordination tasks without formal game rules, reflecting a multimodal intervention structure rather than identical exercise content; SG referred to structured games requiring tactical decision-making and interaction with peers; and SE referred to brief intermittent bouts of moderate-to-vigorous activity, including exercise snacks and HIIT, classified based on shared intermittent and high-intensity characteristics. We acknowledge that ME and SE encompass heterogeneous protocols; these categories were defined to capture shared conceptual features while maintaining network connectivity. These criteria followed the definitions and examples summarized in Supplementary File 3 and aligned with prior classification frameworks reported in the literature. Two reviewers independently assigned interventions to categories, achieving high inter-rater agreement (*k* = 0.89).

At the tertiary level, interventions were quantified by combining modality and dosage, expressed as metabolic equivalent task-minutes per week (METs-min/week). MET values represent energy expenditure calculated by multiplying duration, frequency, and intensity (Ainsworth et al., 2011; Wasfy and Baggish, 2016; Kaminsky, 2014). For instance, an AE intervention could be quantified as 500 METs-min/week. Given typically lower weekly MET values among children and adolescents, exercise doses were categorized in increments of 100 METs ranging from 100 to 1,200 METs-min/week. This approach enhances the connectivity of the analytical network, a practice supported by previous literature (Gallardo-Gómez et al., 2022; Higgins et al., 2012).

### 2.6 Data synthesis

#### 2.6.1 Network meta-analysis

Network plots were generated using Stata version 14 (StataCorp LLC, College Station, Texas, USA) to illustrate trial comparability and ensure feasibility for network meta-analysis. NMA allows the comparison of multiple interventions within a single analytical framework, even in the absence of direct head-to-head trials, enabling comprehensive evaluation of treatment effects. Bayesian network meta-analysis was conducted using R software (version 4.2.2, R Foundation for Statistical Computing) with the “gemtc” and “rjags” packages to estimate comparative treatment effects of PA interventions. Bayesian methods integrated prior information with data through posterior distributions, offering comprehensive data integration advantages over frequentist approaches.

Markov Chain Monte Carlo methods generated posterior samples, and model convergence was assessed via Brooks–Gelman–Rubin plots. Standardized mean difference (SMD) with 95% credible intervals (CrIs) was used as the effect size due to varying scales and outcome units across studies. Mean and SD changes were calculated as:


$${\text{Mean}}_{\text{change}}={\text{Mean}}_{\text{post}}-{\text{Mean}}_{\text{baseline}}$$

$${\mathrm{SD}}_{\text{change}}=\sqrt{\left[({\mathrm{SD}}_{\mathrm{pre}}^{2}+{\mathrm{SD}}_{\text{post}}^{2})-(2\times \text{Corr}\times {\mathrm{SD}}_{\mathrm{pre}}\times {\mathrm{SD}}_{\text{post}})\right]}$$

A random-effects model pooled the data, ranking treatments using the surface under the cumulative ranking curve (SUCRA) (Supplementary file 7).


$$\text{SUCRAi}=\frac{\sum_{i=1}^{k}\mathrm{Cij}}{k-1}$$

A random-effects model was applied for data synthesis, and treatments were ranked using the surface under the cumulative ranking curve (SUCRA) method. Statistical heterogeneity and network inconsistency were assessed through *τ*^2^, *I*^2^ statistics, and design-by-treatment interaction tests (Higgins et al., 2012). SIDE-splitting tests were conducted to compare direct and indirect evidence (Dias et al., 2010) (Supplementary File 5). Publication bias was evaluated using adjusted funnel plots and Egger’s regression test (Chaimani et al., 2013), with *p*-values <0.05 indicating potential bias (Supplementary File 6). To address potential measurement heterogeneity across inhibitory control tasks, we additionally conducted a task-specific sensitivity analysis. Studies using Flanker, Go/No-Go, and Stroop tasks were reanalyzed separately to examine whether intervention rankings remained stable across task types.

#### 2.6.2 Dose–response analysis

Dose–response relationships between exercise dose and inhibitory control were analyzed using the “MBNMAdose” package in R (Mawdsley et al., 2016). Prior to dose–response analysis, the connectedness of interventions was verified to prevent statistical power issues and misinterpretation (Ter Veer et al., 2019). Consistency was confirmed by comparing the deviance information criterion (DIC), residual deviance, and parameter count between consistency and unrelated mean effect models (Pedder et al., 2019) (Supplementary File 9). Transitivity was evaluated using node-splitting methods comparing direct and indirect evidence (van Valkenhoef et al., 2016) (Supplementary File 11).

Linear and nonlinear models, including restricted cubic spline models, were tested to explore potential nonlinear dose–response relationships, with spline knots placed at the 10th, 50th, and 90th percentiles of the dose distribution, following commonly recommended practice for capturing nonlinear exposure-response patterns while maintaining model stability (Evans, 2019; Pedder et al., 2019). These knot placements reflect the underlying distribution of exercise doses in the included trials and were selected to minimize overfitting while allowing sufficient flexibility to model curvature in the dose–response relationship. Nonlinearity was assessed with Wald tests (Hamza et al., 2021) (Supplementary File 8).

## 3 Results

### 3.1 Characteristics of included studies

**Figure 1:**
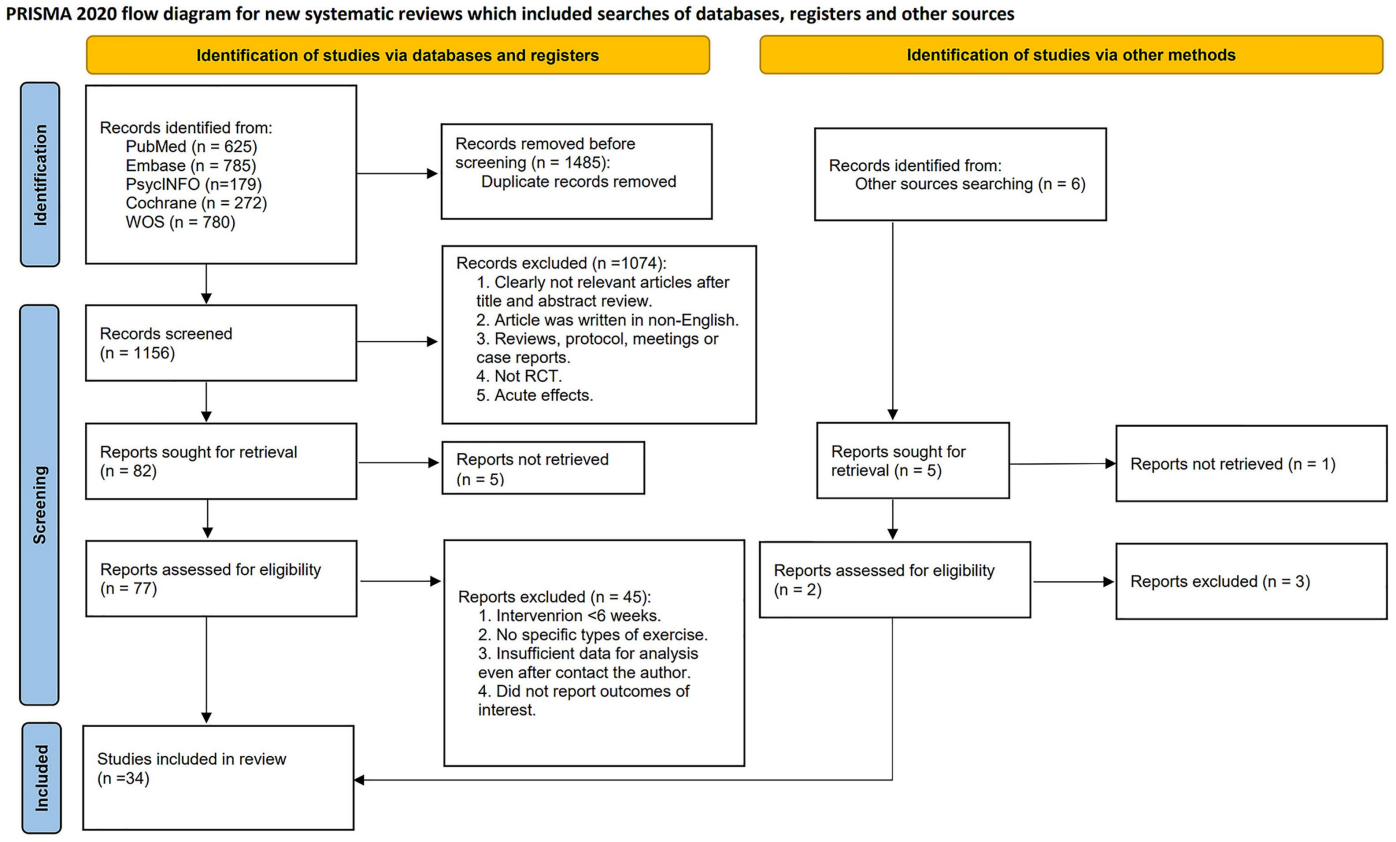
PRISMA flow diagram of the search process for studies. *RCT* randomized controlled trials.

The flow diagram illustrating the study selection process is presented in Figure 1. The initial electronic database search identified 2,641 potentially relevant studies. After screening titles and abstracts, 82 studies were deemed eligible, and full-text articles were retrieved for further review. Following the exclusion of studies not meeting the inclusion criteria, 34 RCTs were included in the meta-analysis (Alesi et al., 2016; Barboza et al., 2021; Beck et al., 2016; Beckmann et al., 2022; Cho et al., 2017; Contreras-Osorio et al., 2022; Drollette et al., 2018; Egger et al., 2019; Gentile et al., 2020; Hillman et al., 2014; Krafft et al., 2014; Kvalø et al., 2017; Leahy et al., 2020; Logan et al., 2021; Lubans et al., 2020; Ludyga et al., 2019a,b, 2021; Mavilidi et al., 2020; Meijer et al., 2020; Mora-Gonzalez et al., 2023; Robinson et al., 2022; Roh et al., 2018; Schmidt et al., 2015; St Laurent et al., 2019; Takehara et al., 2021; Tocci et al., 2022; Torbeyns et al., 2017; van den Berg et al., 2019; Vazou et al., 2020; Veldman et al., 2020; Wassenaar et al., 2021; Zhang et al., 2023; Zinelabidine et al., 2022), involving a total of 23,209 participants, of whom 11,226 were male. The reported mean age of participants was 10.14 years (SD = 2.51), with studies involving children aged 6–12 years accounting for 65.9% of the total sample.

The included studies were categorized into five types of PA interventions and CON (Supplementary File 3). ME interventions were most frequently utilized, accounting for 31.7% of the interventions, while DC were the least common, comprising only 10.0%. Intervention durations per session varied from 4 to 120 min, with sessions lasting 11–30 min and 31–60 min each representing 34.7% of the interventions. Intervention frequencies ranged from one to five times per week, with twice-weekly and five-times-weekly sessions being the most common, each constituting 28.6% of the interventions. Total intervention durations ranged from 4 weeks to 10 months, with interventions lasting 11–20 weeks comprising 40.8% of all included studies (Table 1).

### 3.2 Network meta-analysis

**Figure 2:**
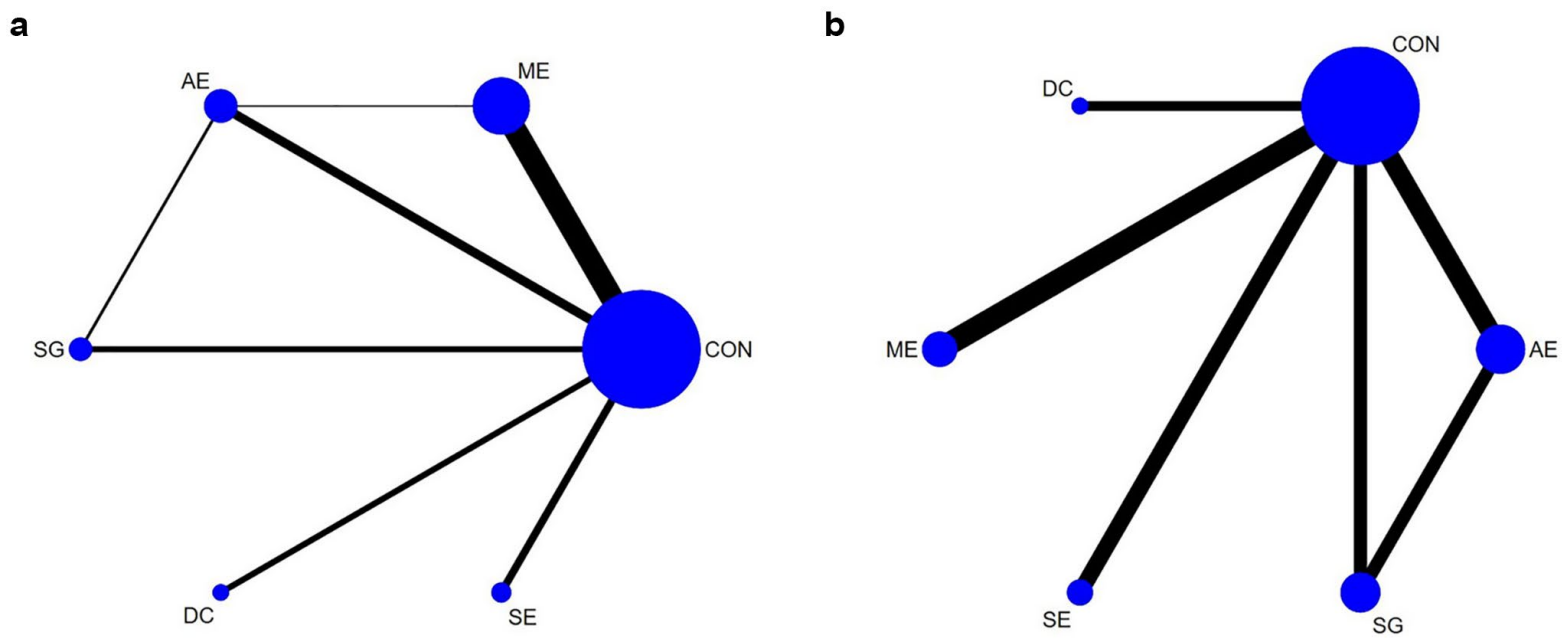
Network plot comparing the effects of different physical activity interventions on inhibitory control outcomes: **(a)** inhibitory control accuracy; **(b)** inhibitory control reaction time.

A total of 31 studies, involving 20,379 participants, reported primary outcomes for inhibition accuracy (Figure 2a); meanwhile, inhibition reaction time outcomes were reported by 21 studies, encompassing 20,621 participants (Figure 2b). Compared to the CON, ME significantly enhanced inhibition accuracy in children and adolescents, yielding a SMD of 0.52 (95% CrI: 0.21–0.82), and demonstrated the highest effectiveness according to the surface under the cumulative ranking curve (SUCRA = 0.81) (Table 2). Conversely, no significant reduction in inhibition reaction time was observed for any of the five PA types compared to CON, although SE exhibited the greatest potential reduction (SUCRA = 0.59) (Table 3).

**Table 2:** The league table of inhibition accuracy.

ME	SG	AE	DC	CON	SE
**ME (0.81)**	NA	−0.01 (−1.21; 1.19)	NA	**0.51 (0.20; 0.82)**	NA
0.03 (−0.62; 0.69)	**SG (0.74)**	0.14 (−0.74; 1.02)	NA	0.40 (−0.21; 1.02)	NA
0.15 (−0.39; 0.70)	0.12 (−0.55; 0.79)	**AE (0.63)**	NA	0.27 (−0.23; 0.77)	NA
0.33 (−0.33; 0.99)	0.30 (−0.53; 1.13)	0.18 (−0.58; 0.93)	**DC (0.44)**	0.18 (−0.40; 0.77)	NA
**0.52 (0.21; 0.82)**	0.48 (−0.11; 1.07)	0.36 (−0.11; 0.84)	0.18 (−0.40; 0.77)	**CON (0.20)**	0.07 (−0.45; 0.59)
0.59 (−0.01; 1.19)	0.55 (−0.23; 1.34)	0.43 (−0.27; 1.14)	0.26 (−0.53; 1.04)	0.07 (−0.45; 0.59)	**SE (0.18)**

All results are presented in the form of SMD (95% CrI). Intervention types are ranked according to the SUCRA for inhibition accuracy with the best from left to right. The results of the network meta-analysis are showed in the lower left part, and results from pairwise comparisons in the upper right half (if available). Cells shown in bold indicate significant results. NA, not available; SMD, standardized mean difference; CrI, credible interval; AE, aerobic exercise; CON, control group; DC, dance; ME, mixed exercise; SE, snack exercise; SG, sports games. The colour shades represent the magnitude and direction of the standardized mean difference (SMD), with darker shades indicating larger effect sizes.

**Table 3:** The league table of inhibition reaction time.

SE	SG	CON	AE	ME	DC
**SE (0.59)**	NA	−0.05 (−0.43; 0.34)	NA	NA	NA
−0.03 (−0.62; 0.55)	**SG (0.53)**	0.03 (−0.43; 0.48)	−0.07 (−0.52; 0.39)	NA	NA
−0.05 (−0.43; 0.34)	−0.01 (−0.45; 0.42)	**CON (0.51)**	−0.01 (−0.40; 0.37)	−0.03 (−0.38; 0.32)	−0.04 (−0.55; 0.46)
−0.06 (−0.60; 0.48)	−0.03 (−0.47; 0.41)	−0.01 (−0.40; 0.37)	**AE (0.48)**	NA	NA
−0.08 (−0.60; 0.44)	−0.05 (−0.61; 0.51)	−0.03 (−0.38; 0.32)	−0.02 (−0.54; 0.50)	**ME (0.45)**	NA
−0.09 (−0.73; 0.54)	−0.06 (−0.73; 0.61)	−0.04 (−0.55; 0.46)	−0.03 (−0.67; 0.60)	−0.01 (−0.63; 0.60)	**DC (0.44)**

All results are presented in the form of SMD (95% CrI). Intervention types are ranked according to the SUCRA for inhibition reaction time with the best from left to right. The results of the network meta-analysis are showed in the lower left part, and results from pairwise comparisons in the upper right half (if available). Cells shown in bold indicate significant results. NA, not available; SMD, standardized mean difference; CrI, credible interval, AE, aerobic exercise; CON, control group; DC, dance, ME, mixed exercise; SE, snack exercise; SG, sports games. The colour shades represent the magnitude and direction of the standardized mean difference (SMD), with darker shades indicating larger effect sizes.

Statistical analyses indicated no significant inconsistency in inhibition accuracy (*Q* = 3.47, df = 4, *τ*^2^ = 0.3096, *p* = 0.4829) or inhibition reaction time (*Q* = 0.40, df = 1, *τ*^2^ = 0.1704, *p* = 0.5296) (Supplementary Table 5). Further, SIDE analysis confirmed the absence of significant inconsistency in these domains. A task-specific sensitivity analysis further showed that the ranking of intervention modalities, including the superiority of mixed exercise for inhibition accuracy, remained consistent when analyses were restricted to Flanker, Go/No-Go, or Stroop tasks, indicating that task heterogeneity did not materially influence the primary findings (Supplementary Table 12).

In addition, publication bias assessment indicated small-study effects for inhibition accuracy but not for inhibition reaction time, as shown by funnel plots and Egger’s test in the Supplementary File 6.

### 3.3 Dose–response relationships

**Figure 3:**
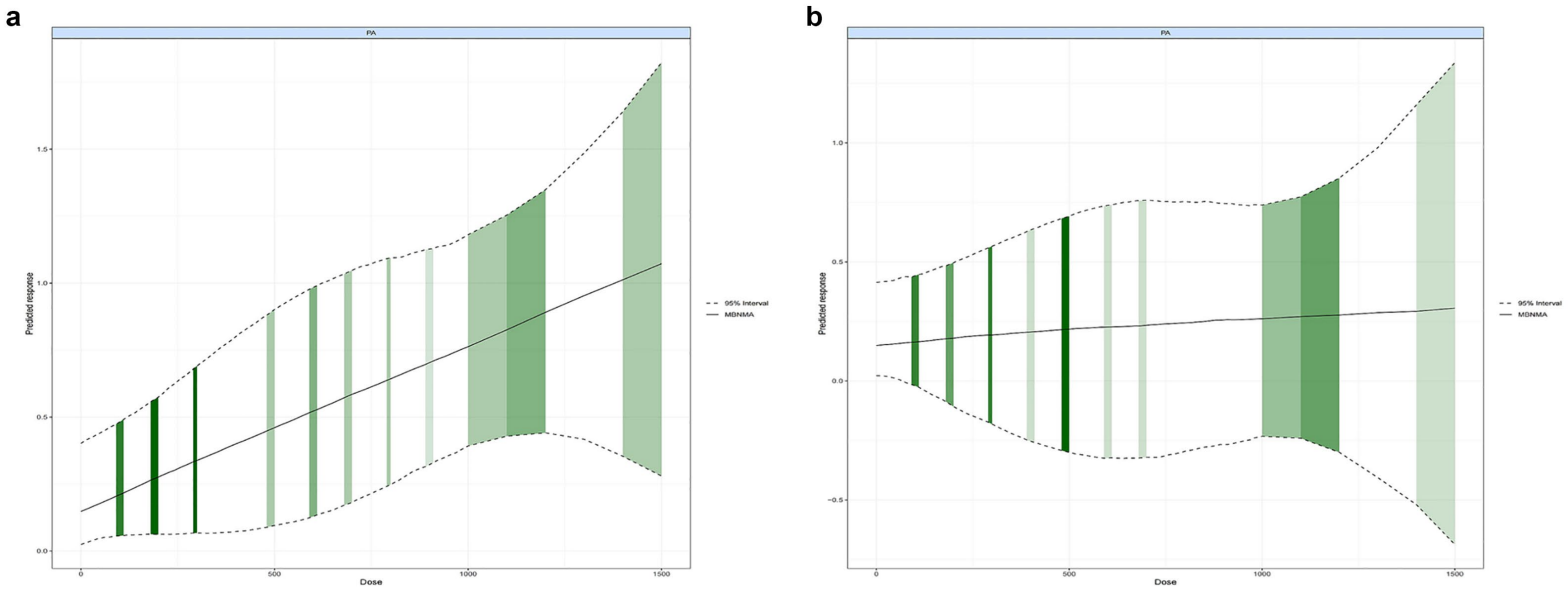
Dose–response relationship between total physical activity levels and inhibitory control outcomes in children and adolescents: **(a)** inhibitory control accuracy; **(b)** inhibitory control reaction time.

Figure 3a illustrates a nonlinear dose–response relationship between total PA dosage and inhibition accuracy, with an effective improvement range extending from 0 to 1,500 METs-min/week. The optimal dosage for enhancing inhibition accuracy in children and adolescents was identified as 1,500 METs-min/week (SMD: 1.08, 95% CrI: 0.29–1.84). Beyond this level, the curve showed little additional gain, indicating a plateau rather than a further increase in effect. This pattern suggests diminishing returns at higher weekly doses. Conversely, Figure 3b demonstrates no significant nonlinear dose–response relationship between total PA dosage and inhibition reaction time, indicating that reaction-time outcomes may be less dose-dependent.

### 3.4 Certainty of the evidence

Across all evaluated outcomes, the overall certainty of evidence was low (Supplementary File 11). Specifically, for the primary outcomes comparing PA interventions with the control group, only 3.6% of the evidence was judged to have moderate certainty; 64.3% of the evidence was rated as low certainty, and 32.1% as very low certainty. When comparing different PA intervention types directly, only 3.8% of the evidence was rated as high certainty, while 69.2% was considered low, and 26.9% very low certainty.

## 4 Discussion

This network meta-analysis integrated data from 34 RCTs, involving a total of 23,209 healthy children and adolescents, to compare the effects of various PA interventions on inhibitory control, as well as to examine the optimal dose–response relationship. Several noteworthy findings emerged from this analysis. First, compared to the CON, ME significantly improved inhibitory control accuracy, and ranked the highest in the surface under the cumulative ranking curve (SUCRA = 0.81). This result aligns with the cognitive stimulation hypothesis, which suggests that more complex physical activities engage a broader range of cognitive processes, providing a stronger challenge for inhibitory control (de Bruijn et al., 2021). The diverse nature of ME, which often involves both aerobic and coordination elements, may provide a more comprehensive cognitive challenge, thus fostering improvements in inhibitory control. Additionally, neuroplasticity theory supports the idea that physically demanding activities, which engage both cognitive and motor functions, promote structural changes in the brain, enhancing cognitive functions like inhibition over time (Augusto-Oliveira et al., 2023). Second, a significant nonlinear dose–response relationship was observed between total PA dosage and inhibitory control accuracy, with no such effect found for inhibition reaction time. Specifically, when the total PA dosage ranged from 0 to 1,500 METs-min/week, inhibitory control accuracy improved substantially, with the optimal dosage identified as 1,500 METs-min/week. From a practical perspective, this dosage is broadly equivalent to achieving approximately 60 min per day of moderate-to-vigorous physical activity across the week, which is consistent with current international physical activity recommendations for children and adolescents. This finding highlights the critical role of exercise dosage in enhancing inhibitory control, underscoring that the benefits of PA are dose-dependent. However, no significant dose-related improvements were seen in inhibition reaction time, indicating that reaction time may be influenced by other factors beyond just the volume of exercise. The consistent optimal dosage across overall PA reinforces the importance of intensity, frequency, and duration in determining the effectiveness of exercise interventions aimed at enhancing cognitive function. This dose–response relationship also resonates with the embodied cognition theory, which suggests that physical activity enhances cognition by promoting bodily engagement with the environment, facilitating the brain’s development through regular, structured movement (Ravn, 2022). Nevertheless, these findings should be interpreted in light of the overall certainty of evidence supporting the network estimates.

Inhibitory control accuracy plays a crucial role in children’s ability to regulate their impulses and engage in goal-directed behaviors. This aspect of executive function is essential for academic success, social interactions, and emotional regulation. Unlike reaction time, which reflects the speed of response inhibition, accuracy in inhibitory control indicates how well children can suppress prepotent responses in favor of more appropriate or socially acceptable ones (Dumont et al., 2022). Therefore, improving inhibitory control accuracy has significant implications for enhancing self-regulation and reducing behavior problems in children and adolescents. Currently, many children and adolescents struggle with underdeveloped inhibitory control, which may manifest as impulsive behavior, difficulty with attention, and challenges in school settings (Fosco et al., 2019). As inhibitory control is still maturing during this developmental phase, targeted interventions can play a vital role in accelerating the refinement of this cognitive function. However, interventions are often required to be specifically tailored to address this developmental gap, and not all methods are equally effective.

This study’s first key finding emphasizes that ME is the most effective PA intervention for improving inhibitory control accuracy in children and adolescents. ME outperformed all other exercise modalities in this study, as evidenced by its highest ranking in the cumulative ranking curve (SUCRA = 0.81) and a significant improvement in inhibitory control accuracy (SMD = 0.52, 95% CrI: 0.21–0.82). This result aligns with previous findings suggesting that multi-component or complex motor activities yield greater benefits for cognitive functions compared to simpler forms of exercise. For example, ball games, which require coordination, strategic thinking, and social interaction, have been reported to significantly impact cognitive function, including inhibitory control (Benzing and Schmidt, 2017). However, unlike prior studies that emphasized ball games, our analysis highlights that ME, which likely integrates multiple exercise forms, stands out as the most effective. Several potential mechanisms may explain why ME is particularly effective in improving inhibitory control accuracy. ME typically combines aerobic, strength, and coordination exercises, providing a multifaceted challenge to the brain. The integration of cognitive and motor tasks during ME interventions may stimulate neural pathways involved in self-regulation and executive function (Shi et al., 2022). Moreover, research on neurodevelopment suggests that physical activity, particularly exercises that engage both the brain and body, enhances neuroplasticity, which plays a critical role in cognitive improvements during developmental stages. The intensity and variety inherent in ME interventions may better align with the evolving neurocognitive needs of children and adolescents, promoting greater development of inhibitory control accuracy (Donnelly et al., 2016). Furthermore, the complex motor demands of ME activities—requiring not only physical coordination but also strategic planning and decision-making—may create more opportunities for the brain to practice inhibitory control in varied contexts (Formenti et al., 2021). Additionally, the potential social aspects of ME, such as those found in group-based activities like team sports, could further enhance inhibitory control by fostering communication, teamwork, and emotional regulation skills (Yao et al., 2024). These social and cognitive demands are crucial for reinforcing inhibitory control accuracy, as they require ongoing self-monitoring and suppression of inappropriate responses in real-time. Importantly, although ME showed the most favorable ranking, most direct and indirect comparisons contributing to this estimate were supported by low or very low certainty evidence according to the CINeMA assessment, which warrants cautious interpretation of the magnitude of effects.

Interestingly, SE ranked last in improving inhibition accuracy, but it performed the best in reducing inhibition reaction time. This contrasting effect highlights the complexity of how different exercise modalities influence various aspects of inhibitory control. While SE excels in improving speed, it appears to have limited effects on accuracy, suggesting that the specific characteristics of the exercise type may engage distinct cognitive processes. SE’s strong effect on reaction time, paired with its low ranking in accuracy, underscores its potential to enhance the speed at which children and adolescents suppress responses. To further interpret these modality-specific differences, it is important to consider the underlying neural mechanisms. ME is likely to stimulate frontoparietal and frontostriatal networks that support sustained top-down inhibitory control, whereas SE may preferentially enhance neural pathways associated with rapid motor preparation and response initiation. Evidence indicates that complex, cognitively engaging activities enhance prefrontal activation and strengthen functional connectivity within executive control networks, while also increasing neurotrophic factors such as BDNF that promote long-term neuroplasticity (Wu et al., 2022). In contrast, short high-intensity bouts characteristic of SE are more strongly associated with transient increases in catecholamines and cerebral blood flow, which primarily facilitate processing speed rather than accuracy-dependent inhibitory regulation (Shao et al., 2023). These distinct neurophysiological pathways provide a plausible explanation for why ME improved accuracy and SE improved reaction time.

This finding is consistent with previous studies indicating that high-intensity intermittent exercises, such as SE or high-intensity interval training (HIIT), tend to improve cognitive processing speed but may not significantly enhance accuracy in tasks requiring inhibition (Gilson et al., 2023). The nature of SE, which includes snack exercises and HIIT, likely contributes to its effect on reaction time. These exercises involve short bursts of intense activity that enhance cardiovascular fitness and neural efficiency, which may improve speed and agility in decision-making processes (Wang et al., 2025). However, the brief nature of SE may not provide the sustained cognitive engagement necessary to improve inhibition accuracy. From a neurodevelopmental perspective, the prefrontal cortex and basal ganglia, which are involved in motor control and executive function, continue to mature throughout childhood and adolescence. SE’s short, high-intensity bursts likely engage the brain areas responsible for rapid response inhibition and coordination, improving processing speed. However, accuracy in inhibition requires more continuous, complex cognitive control, which may be better addressed by interventions that involve longer durations of cognitive engagement and varied motor tasks, such as ME or aerobic exercises (Wassenaar et al., 2021). SE demonstrates significant improvements in inhibition reaction time, but its limited impact on inhibition accuracy can be attributed to the nature of the exercise modality. This finding highlights the need for targeted interventions that balance both speed and accuracy in improving inhibitory control. Future research should explore the neural mechanisms underlying these effects and refine SE protocols to optimize both reaction time and accuracy outcomes.

In addition, the interpretation of these findings should consider the heterogeneity of inhibitory control tasks across the included studies. Although all tasks were designed to assess inhibition, paradigms such as the Flanker, Go/No-Go, and Stroop tasks differ in stimulus complexity, cognitive load, and response modality, which may influence their sensitivity to physical activity interventions. To address this, we conducted a task-specific sensitivity analysis in which studies using each paradigm were analyzed separately. The ranking of intervention modalities, including the superiority of mixed exercise for inhibition accuracy, remained stable across all task subsets, indicating that the primary conclusions were robust to measurement heterogeneity. Moreover, the differential responses observed for inhibition accuracy and reaction time likely reflect distinct underlying mechanisms: accuracy depends more on sustained top-down control, whereas reaction time is more closely related to rapid motor processing and speeded response selection (Anderson et al., 2025). These differences may explain why physical activity consistently improved accuracy but did not show a clear dose-related effect on reaction time.

The present study conducted a dose–response analysis to explore the relationship between PA dosage and inhibitory control, focusing on both inhibition accuracy and reaction time. The results showed that total PA dosage was positively associated with improvements in inhibition accuracy, with the optimal effect observed at 1500 METs-min/week. The restricted cubic spline model used in this study placed knots at the 10th, 50th, and 90th percentiles of the observed dose distribution, a placement strategy commonly recommended for identifying nonlinear exposure-response patterns while maintaining model stability. This suggests that PA at 1,500 METs-min/week is the most effective dosage for enhancing inhibition accuracy. Beyond this point, the curve leveled off, indicating a plateau rather than continued improvement, which is consistent with a diminishing-returns pattern often seen in exercise-cognition research. In contrast, no significant dose-effect relationship was found for inhibition reaction time, indicating that reaction time may not be as closely linked to PA dosage as inhibition accuracy. These findings are consistent with previous studies that have reported dose-dependent effects of PA on cognitive functions, particularly executive control (Wang et al., 2024). However, this study is the first to clearly identify 1,500 METs-min/week as the optimal PA dosage for improving inhibition accuracy, underscoring the importance of dosage in influencing cognitive outcomes. The peak improvement in inhibition accuracy at 1,500 METs-min/week can be attributed to the optimal balance between exercise intensity and duration, which supports neuroplasticity in brain regions responsible for executive functions, such as the prefrontal cortex and basal ganglia. Previous research has shown that moderate levels of PA promote synaptic growth and enhance the functioning of these brain regions, which are crucial for inhibitory control (Stanmore et al., 2017). This level of PA may also facilitate cognitive adaptations that improve the brain’s ability to suppress inappropriate responses while maintaining efficient cognitive processing. In interpreting the dose–response findings, we categorized PA dosage into 100 METs-min/week increments to maintain adequate network connectivity across trials with heterogeneous reporting of duration, frequency, and intensity. The nonlinear pattern and the identification of 1,500 METs-min/week as the optimal range were consistent when using alternative cut points, and the spline-based analysis treated dose as a continuous variable, which reduces information loss and supports the robustness of this estimate. In addition, the clinical relevance of these effects should be noted. The moderate improvement in inhibition accuracy associated with mixed exercise is likely to reflect better attentional control and behavioral regulation in everyday school contexts. However, the extent to which these cognitive gains translate into measurable academic or behavioral outcomes still needs to be confirmed in future trials that incorporate educational or behavioral follow-up indicators.

This study offers several notable strengths. It provides a comprehensive synthesis of RCTs using NMA to compare multiple PA modalities and their effects on inhibitory control, and it incorporates a dose–response analysis that identifies 1,500 METs-min/week as a potentially optimal dosage for improving inhibition accuracy in children and adolescents. Several limitations should be considered when interpreting these findings. First, the included trials showed substantial heterogeneity in PA type, duration, intensity, and participant characteristics, which may reduce the generalizability of comparisons between modalities. This heterogeneity was particularly relevant for complex exercise categories such as mixed exercise and exercise snacks, which encompassed diverse intervention components despite shared conceptual features. Second, children with developmental disorders or physical impairments were largely excluded, and most studies did not report age-stratified results across the 6–18-year range, so the findings represent average effects and cannot clarify age-specific responses. Third, the certainty of evidence was predominantly low or very low because of within-study bias, imprecision, heterogeneous assessment tools, and non-standardized reporting of exercise protocols. Information on intervention fidelity and adherence was also limited, meaning that variation in delivery quality and participant engagement may have influenced effect sizes. In addition, most trials used passive control conditions, and sex-stratified outcomes were rarely reported, which prevented formal examination of sex-related differences. Finally, although an optimal PA dose was identified at the behavioral level, the underlying neurobiological mechanisms remain unclear, and future studies combining rigorous intervention reporting with neuroimaging or biomarker approaches are needed to clarify how specific PA types and doses affect neuroplasticity and inhibitory control.

## 5 Conclusion

This network meta-analysis provides compelling evidence on the effectiveness of PA in enhancing inhibitory control in children and adolescents. Among the various exercise modalities, ME emerged as the most effective intervention for improving inhibition accuracy. Furthermore, the study identified a significant dose–response relationship between total PA dosage and inhibition accuracy, reinforcing the critical role of PA intensity, frequency, and duration in cognitive outcomes. While no significant dose-related improvements were observed for inhibition reaction time, SE demonstrated the greatest improvement in speed. These findings underscore the importance of selecting appropriate PA types and dosages for maximizing cognitive benefits. Future research should focus on exploring the underlying neural mechanisms and the applicability of these interventions in diverse populations.

## Data Availability

The original contributions presented in the study are included in the article/Supplementary material, further inquiries can be directed to the corresponding author.

## Glossary

PA: physical activity
EF: executive functions
MVPA: moderate to vigorous physical activity
RCT: randomized controlled trials
METs: metabolic equivalent of tasks
AE: aerobic exercise
DC: dance
ME: mixed exercise
SE: snack exercise
SG: sports game
CON: control group
SMD: standardized mean differences
CrI: credible interval
DIC: deviance information criterion
CINeMA: Confidence in Network Meta-Analysis
MBNMA: model-based network meta-analysis
PRISMA: Preferred Reporting Items for Systematic Reviews and Meta-Analyses
BMI: body mass index
SD: standard deviation
SUCRA: surface under the cumulative ranking curve
