# Supplementary material for: Optimal physical activity type and dosage for improving inhibitory control in children and adolescents: a dose–response network meta-analysis
Source: Front Psychol. 2026 Mar 12;17:1702811. doi: 10.3389/fpsyg.2026.1702811 (PMC13017248; doi:10.3389/fpsyg.2026.1702811)
Supplement: Supplementary file 1 [file Table_1.DOCX]

**Supplementary**

Table of Contents:

[Supplementary 1: PRISMA Checklist 1](#_Toc534204563)

[Supplementary 2: Search Strategy 4](#_Toc1894445881)

[Supplementary 3: Definitions and examples of physical activity types 6](#_Toc882098351)

[Supplementary 4: Risk of Bias](#_Toc1262935083) 8

[Supplementary 5: Evaluation of heterogeneity and inconsistency 10](#_Toc829414178)

[Supplementary 6: Publication bias](#_Toc431254745) 12

[Supplementary 7: Forest plot](#_Toc341190590) 13

[Supplementary 8: Assessment of Model Consistency](#_Toc608908640) 15

[Supplementary 9: Node-splitting analysis of inconsistency](#_Toc1167934525) 16

[Supplementary 10: Non-linear functions and models fit comparison](#_Toc1475028095) 21

[Supplementary 11: CINeMA outcomes](#_Toc245971697) 26

[Supplementary 12: Sensitivity analysis by inhibitory control task type](#_Toc1475028095) 30

**Supplementary 1: PRISMA Checklist**

| Item # | Checklist Item | Reported on Page # |
| --- | --- | --- |
|  |  | 1 |
| 1 | Identify the report as a systematic review incorporating a network meta-analysis (or related form of meta-analysis). | 1 |
| 2 | Provide a structured summary including, as applicable:  **Background:** main objectives  **Methods:** data sources; study eligibility criteria, participants, and interventions; study appraisal; and *synthesis methods, such as network meta-analysis.*  **Results:** number of studies and participants identified; summary estimates with corresponding confidence/credible intervals; treatment rankings may also be discussed. Authors may choose to summarize pairwise comparisons against a chosen treatment included in their analyses for brevity.  **Discussion/Conclusions:** limitations; conclusions and implications of findings.  **Other:** primary source of funding; systematic review registration number with registry name. | 1-2 |
| 3 | Describe the rationale for the review in the context of what is already known*, including mention of why a network meta-analysis has been conducted.* | 3 |
| 4 | Provide an explicit statement of questions being addressed, with reference to participants, interventions, comparisons, outcomes, and study design (PICOS). | 3-4 |
| 5 | Indicate whether a review protocol exists and if and where it can be accessed (e.g., Web address); and, if available, provide registration information, including registration number. | 4 |
| 6 | Specify study characteristics (e.g., PICOS, length of follow-up) and report characteristics (e.g., years considered, language, publication status) used as criteria for eligibility, giving rationale. *Clearly describe eligible treatments included in the treatment network, and note whether any have been clustered or merged into the same node (with justification).* | 4-5 |
| 7 | Describe all information sources (e.g., databases with dates of coverage, contact with study authors to identify additional studies) in the search and date last searched. | 4 |
| 8 | Present full electronic search strategy for at least one database, including any limits used, such that it could be repeated. | Supplementary 2 |
| 9 | State the process for selecting studies (i.e., screening, eligibility, included in systematic review, and, if applicable, included in the meta-analysis). | 5 |
| 10 | Describe method of data extraction from reports (e.g., piloted forms, independently, in duplicate) and any processes for obtaining and confirming data from investigators. | 5 |
| 11 | List and define all variables for which data were sought (e.g., PICOS, funding sources) and any assumptions and simplifications made. | 5 |
| S1 | Describe methods used to explore the geometry of the treatment network under study and potential biases related to it. This should include how the evidence base has been graphically summarized for presentation, and what characteristics were compiled and used to describe the evidence base to readers. | Supplementary 6 |
| 12 | Describe methods used for assessing risk of bias of individual studies (including specification of whether this was done at the study or outcome level), and how this information is to be used in any data synthesis. | 6, Supplementary 8 and Figure 5 |
| 13 | State the principal summary measures (e.g., risk ratio, difference in means). Also describe the use of additional summary measures assessed, such as treatment rankings and surface under the cumulative ranking curve (SUCRA) values, as well as modified approaches used to present summary findings from meta-analyses. | 7 |
| 14 | Describe the methods of handling data and combining results of studies for each network meta-analysis. This should include, but not be limited to:   - Handling of multi-arm trials; - Selection of variance structure; - Selection of prior distributions in Bayesian analyses; and - Assessment of model fit. | 7 |
| S2 | Describe the statistical methods used to evaluate the agreement of direct and indirect evidence in the treatment network(s) studied. Describe efforts taken to address its presence when found. | 8 |
| 15 | Specify any assessment of risk of bias that may affect the cumulative evidence (e.g., publication bias, selective reporting within studies). | 7 |
| 16 | Describe methods of additional analyses if done, indicating which were pre-specified. This may include, but not be limited to, the following:   - Sensitivity or subgroup analyses; - Meta-regression analyses; - Alternative formulations of the treatment network; and - Use of alternative prior distributions for Bayesian analyses (if applicable). | NA |
| 17 | Give numbers of studies screened, assessed for eligibility, and included in the review, with reasons for exclusions at each stage, ideally with a flow diagram. | Figure 1 |
| S3 | Provide a network graph of the included studies to enable visualization of the geometry of the treatment network. | Figure 3 |
| S4 | Provide a brief overview of characteristics of the treatment network. This may include commentary on the abundance of trials and randomized patients for the different interventions and pairwise comparisons in the network, gaps of evidence in the treatment network, and potential biases reflected by the network structure. | Supplementary 9 |
| 18 | For each study, present characteristics for which data were extracted (e.g., study size, PICOS, follow-up period) and provide the citations. | Figure 1， and Supplementary 2 |
| 19 | Present data on risk of bias of each study and, if available, any outcome level assessment. | Figure 2, and Supplementary 8 |
| 20 | For all outcomes considered (benefits or harms), present, for each study: 1) simple summary data for each intervention group, and 2) effect estimates and confidence intervals. *Modified approaches may be needed to deal with information from larger networks.* | NA |
| 21 | Present results of each meta-analysis done, including confidence/credible intervals. In larger networks, authors may focus on comparisons versus a particular comparator (e.g. placebo or standard care), with full findings presented in an appendix. League tables and forest plots may be considered to summarize pairwise comparisons. If additional summary measures were explored (such as treatment rankings), these should also be presented. | 9-11 and Supplementary 9 |
| S5 | Describe results from investigations of inconsistency. This may include such information as measures of model fit to compare consistency and inconsistency models, *P* values from statistical tests, or summary of inconsistency estimates from different parts of the treatment network. | Supplementary 5 |
| 22 | Present results of any assessment of risk of bias across studies for the evidence base being studied. | 8-9 |
| 23 | Give results of additional analyses, if done (e.g., sensitivity or subgroup analyses, meta-regression analyses*, alternative network geometries studied, alternative choice of prior distributions for Bayesian analyses,* and so forth). | 9, and Table2, and Supplementary 6, and Supplementary 8 |
| 24 | Summarize the main findings, including the strength of evidence for each main outcome; consider their relevance to key groups (e.g., healthcare providers, users, and policy-makers). | 12 |
| 25 | Discuss limitations at study and outcome level (e.g., risk of bias), and at review level (e.g., incomplete retrieval of identified research, reporting bias). *Comment on the validity of the assumptions, such as transitivity and consistency. Comment on any concerns regarding network geometry (e.g., avoidance of certain comparisons).* | 12-16 |
| 26 | Provide a general interpretation of the results in the context of other evidence, and implications for future research. | 16 |
| 27 | Describe sources of funding for the systematic review and other support (e.g., supply of data); role of funders for the systematic review. This should also include information regarding whether funding has been received from manufacturers of treatments in the network and/or whether some of the authors are content experts with professional conflicts of interest that could affect use of treatments in the network. | 17 |

PICOS = population, intervention, comparators, outcomes, study design.

**Supplementary 2: Search Strategy**

***Search Strategy:***

| Search number | Query |
| --- | --- |
| 17 | (((((((((((child[MeSH Terms]) OR (adolescent[MeSH Terms])) OR (children[Title/Abstract])) OR (adolescen*[Title/Abstract])) OR (pupils[Title/Abstract])) OR (preadolescent[Title/Abstract])) OR (youth[Title/Abstract])) OR (juvenile[Title/Abstract])) OR (teenager*[Title/Abstract])) AND ((((((((exercise[MeSH Terms]) OR ("physical activity"[Title/Abstract])) OR (training[Title/Abstract])) OR (workout[Title/Abstract])) OR (sport*[Title/Abstract])) OR (jogging[Title/Abstract])) OR ("resistance training"[Title/Abstract])) OR ("aerobic exercise"[Title/Abstract]))) AND (((((executive function[MeSH Terms]) OR ("executive function*"[Title/Abstract])) OR (inhibition[Title/Abstract])) OR (inhibitory[Title/Abstract])) ) AND ((randomized controlled trial[pt] OR controlled clinical trial[pt] OR randomized[tiab] OR placebo[tiab] OR drug therapy[sh] OR randomly[tiab] OR trial[tiab] OR groups[tiab]) NOT (animals[mh] NOT humans[mh])) |
| 16 | (randomized controlled trial[pt] OR controlled clinical trial[pt] OR randomized[tiab] OR placebo[tiab] OR drug therapy[sh] OR randomly[tiab] OR trial[tiab] OR groups[tiab]) NOT (animals[mh] NOT humans[mh]) |
| 15 | humans[mh] |
| 14 | animals[mh] |
| 13 | randomized controlled trial[pt] OR controlled clinical trial[pt] OR randomized[tiab] OR placebo[tiab] OR drug therapy[sh] OR randomly[tiab] OR trial[tiab] OR groups[tiab] |
| 12 | (((((((executive function[MeSH Terms]) OR ("executive function*"[Title/Abstract])) OR (inhibition[Title/Abstract])) OR (inhibitory[Title/Abstract]) |
| 11 | ("executive function*"[Title/Abstract]) OR (inhibition[Title/Abstract]) OR (inhibitory[Title/Abstract]) |
| 10 | executive function[MeSH Terms] |
| 9 | (((((((exercise[MeSH Terms]) OR (resistance training[MeSH Terms]) OR (Yoga[MeSH Terms]) OR ("physical activity"[Title/Abstract])) OR (training[Title/Abstract])) OR (workout[Title/Abstract])) OR (sport*[Title/Abstract])) OR (jogging[Title/Abstract])) OR ("resistance training"[Title/Abstract])) OR ("aerobic exercise"[Title/Abstract]) |
| 8 | ("physical activity"[Title/Abstract]) OR (training[Title/Abstract]) OR (workout[Title/Abstract]) OR (sport*[Title/Abstract]) OR (jogging[Title/Abstract]) OR ("resistance training"[Title/Abstract]) OR ("aerobic exercise"[Title/Abstract]) |
| 7 | Yoga[MeSH Terms] |
| 6 | Resistance training[MeSH Terms] |
| 5 | (exercise[MeSH Terms] |
| 4 | ((((((((child[MeSH Terms]) OR (adolescent[MeSH Terms])) OR (children[Title/Abstract])) OR (adolescen*[Title/Abstract])) OR (pupils[Title/Abstract])) OR (preadolescent[Title/Abstract])) OR (youth[Title/Abstract])) OR (juvenile[Title/Abstract])) OR (teenager*[Title/Abstract]) |
| 3 | (children[Title/Abstract]) OR (adolescen*[Title/Abstract]) OR (pupils[Title/Abstract]) OR (preadolescent[Title/Abstract]) OR (youth[Title/Abstract]) OR (juvenile[Title/Abstract]) OR (teenager*[Title/Abstract]) |
| 2 | Adolescent[MeSH Terms] |
| 1 | Child[MeSH Terms] |

# Supplementary file 3: Definitions and examples of physical activity types

| **abbreviation** | **Full name** | **Definitions** | **Examples** |
| --- | --- | --- | --- |
| AE | Aerobic Exercise | Aerobic exercise aims to enhance cardiorespiratory function through sustained, rhythmic, and moderate-intensity physical activities. Predominantly reliant on oxygen for energy production, these exercises typically have an extended duration^1^. | Jogging, jumping rope, relay races, treadmill training and circuit training, etc. |
| CON | Control  Group | Regular physical education. | Regular teaching programs on physical activity. |
| DC | Dance | Dance constitutes a choreographed form of physical activity, synchronized to musical rhythms and compositions. | Aerobic dancing to specific rhythmic patterns. |
| ME | Mixed Exercise | Mixed exercise integrates diverse modalities of physical activity, encompassing cardiovascular training, resistance exercises, and various game-based activities, to offer a well-rounded workout regimen. This multifaceted approach is frequently employed in cross-training programs to optimize fitness outcomes. | Starting with a cardio warm-up, followed by a series of resistance exercises such as squats and lunges, and concluding with sports games to develop skills and team coordination. |
| SE | Snack  Exercise | Snacks Exercise comprises short-duration, intermittent physical activities interspersed throughout the day, primarily aimed at disrupting prolonged sedentary periods and promoting health^2^. These exercises are characterized by moderate to high intensity, reaching up to maximal effort, with each session lasting no more than 10 minutes^3^. They demand minimal equipment and environmental requirements, making them highly accessible and convenient for incorporation into daily routines. | High-Intensity Interval Training (HIIT) sessions that can be quickly completed; classroom breaks designed for brief physical engagement, such as stretching or quick aerobic activities, to rejuvenate focus and energy. |
| SG | Sports  Game | Sports game is organized physical activities designed to foster skill acquisition and physical engagement through structured play^4,5^. These activities seamlessly integrate elements of gameplay, competition, and skill progression, tailored to align with the developmental stages of young individuals. Specifically crafted to enhance physical conditioning, social competencies, collaborative teamwork, and strategic cognition, these games serve as a foundational platform for holistic growth in children and adolescents. | Mini soccer modifies traditional football rules to accommodate younger players, featuring smaller teams and fields to match their physical dimensions and skill levels. Tag games, known for bolstering speed, nimbleness, and interpersonal interaction, engage participants in dynamic movement and social engagement. |

**Reference**

1. Thompson PD, Arena R, Riebe D, Pescatello LS. ACSM's new preparticipation health screening recommendations from ACSM's guidelines for exercise testing and prescription, ninth edition. Current sports medicine reports. 2013;12(4):215-217.

2. Islam H, Gibala MJ, Little JP. Exercise Snacks: A Novel Strategy to Improve Cardiometabolic Health. Exerc Sport Sci Rev. 2022 Jan 1;50(1):31-37. doi: 10.1249/JES.0000000000000275. PMID: 34669625.

3. Ahmadi MN, Hamer M, Gill JMR, Murphy M, Sanders JP, Doherty A, Stamatakis E. Brief bouts of device-measured intermittent lifestyle physical activity and its association with major adverse cardiovascular events and mortality in people who do not exercise: a prospective cohort study. Lancet Public Health. 2023 Oct;8(10):e800-e810. doi: 10.1016/S2468-2667(23)00183-4. PMID: 37777289.

4. Varghese M, Ruparell S, LaBella C. Youth Athlete Development Models: A Narrative Review. Sports Health. 2022 Jan-Feb;14(1):20-29. doi: 10.1177/19417381211055396. Epub 2021 Nov 10. PMID: 34758649; PMCID: PMC8669922.

5. Whitley MA, Massey WV, Camiré M, Boutet M, Borbee A. Sport-based youth development interventions in the United States: a systematic review. BMC Public Health. 2019 Jan 18;19(1):89. doi: 10.1186/s12889-019-6387-z. PMID: 30658607; PMCID: PMC6339434.

# Supplementary 4: Risk of Bias

## Table 4.1 The risk of bias assessment for the individual included studies

| **Study** | Randomization process | Deviations from intended interventions | Missing outcome data | Measurement of the outcome | Selection of the reported result | Overall Bias |
| --- | --- | --- | --- | --- | --- | --- |
| Alesi et al. (2016) | Low | Some concerns | Low | Low | Low | Some concerns |
| Barboza et al. (2021) | Some concerns | Low | Low | Low | Low | Some concerns |
| Beck et al. (2016) | Some concerns | Low | Low | Low | Low | Some concerns |
| Beckmann et al. (2022) | Low | Low | Low | Low | Low | Low |
| Cho et al. (2017) | Low | Low | Low | Low | Low | Low |
| Contreras-Osorio et al. (2022) | Low | Low | Low | Low | Low | Low |
| Drollette et al. (2011) | Low | Low | Low | Low | Low | Low |
| Egger et al. (2019) | Low | Low | Low | Low | Low | Low |
| Gentile et al. (2020) | Some concerns | Some concerns | Some concerns | Low | Low | Some concerns |
| Hillman et al. (2014) | Low | Low | High | Low | Low | High |
| Krafft et al. (2014) | Low | Low | Low | Low | Low | Low |
| Kvalø et al. (2017) | Low | Low | Some concerns | Low | Low | Some concerns |
| Leahy et al. (2020) | Low | Low | Low | Low | Low | Low |
| Logan et al. (2020) | Low | Low | Low | Low | Low | Low |
| Lubans et al. (2020) | Low | Low | High | Low | Low | High |
| Ludyga et al. (2019, a) | Low | Low | Low | Low | Low | Low |
| Ludyga et al. (2019, b) | Low | Low | Low | Low | Low | Low |
| Ludyga et al. (2021) | Low | High | Low | Some concerns | Low | High |
| Mavilidi et al. (2020) | Low | Low | Low | Low | Low | Low |
| Meijer et al. (2021) | Low | Low | Some concerns | Low | Low | Some concerns |
| Mora‐Gonzalez et al. (2023) | Low | Low | Low | Low | Low | Low |
| Robinson et al. (2022) | Low | Low | Some concerns | Low | Low | Some concerns |
| Roh et al. (2018) | Low | Low | Low | Low | Low | Low |
| Schmidt et al. (2015) | Low | Low | Low | Low | Low | Low |
| St Laurent et al. (2019) | Some concerns | Some concerns | Some concerns | Low | Low | Some concerns |
| Takehara et al. (2021) | Low | Low | High | Low | Low | High |
| Tocci et al. (2022) | Low | Low | Low | Low | Low | Low |
| Torbeyns et al. (2017) | Some concerns | Low | Low | Low | Low | Some concerns |
| van den Berg et al. (2019) | Low | Low | Low | Low | Low | Low |
| Vazou et al. (2020) | Low | Low | Low | Low | Low | Low |
| Veldman et al. (2020) | Low | Low | High | Low | Low | High |
| Wassenaar et al. (2021) | Low | Low | Low | Low | Low | Low |
| Zhang et al.(2023) | Some concerns | Low | Low | Low | Low | Some concerns |
| Zinelabidine et al. (2022) | Low | Low | Low | Low | Low | Low |

# Supplementary 5: Evaluation of heterogeneity and inconsistency

**Table 5.1: Quantifying heterogeneity**

| **Outcomes** | **τ^2^** | **Q** | **df** | **P** | **I^2^** | **Heterogeneity assessment** |
| --- | --- | --- | --- | --- | --- | --- |
| Inhibition accuracy | 0.3076 | 345.25 | 29 | <0.0001 | 91.6% | High |
| Inhibition reaction time | 0.1676 | 215.60 | 20 | <0.0001 | 90.7% | High |

**Table 5.2 Summary of the global inconsistency and SIDE splitting results**

| Outcomes | the Design-by-Treatment test | | | |
| --- | --- | --- | --- | --- |
|  | Q | df | τ^2^ | p-value |
| Inhibition accuracy | 3.47 | 4 | 0.3096 | 0.4829 |
| Inhibition reaction time | 0.40 | 1 | 0.1704 | 0.5296 |

**Table 5.3.1 Details of SIDE splitting results (Inhibition accuracy)**

| **comparison** | **k** | **prop** | **nma** | **direct** | **indir.** | **Diff** | **z** | **p-value** |
| --- | --- | --- | --- | --- | --- | --- | --- | --- |
| AE vs CON | 6 | 0.91 | 0.3614 | 0.271 | 1.2798 | -1.0088 | -1.19 | 0.2357 |
| AE vs DC | 0 | 0 | 0.1766 | NA | 0.1766 | NA | NA | NA |
| AE vs ME | 1 | 0.21 | -0.1541 | 0.0076 | -0.1968 | 0.2044 | 0.3 | 0.7663 |
| AE vs SE | 0 | 0 | 0.4331 | NA | 0.4331 | NA | NA | NA |
| AE vs SG | 2 | 0.57 | -0.1192 | -0.14 | -0.0913 | -0.0487 | -0.07 | 0.9436 |
| DC vs CON | 4 | 1 | 0.1848 | 0.1848 | NA | NA | NA | NA |
| ME vs CON | 15 | 0.98 | 0.5155 | 0.5091 | 0.8875 | -0.3784 | -0.31 | 0.7529 |
| SE vs CON | 5 | 1 | -0.0717 | -0.0717 | NA | NA | NA | NA |
| SG vs CON | 4 | 0.91 | 0.4805 | 0.4017 | 1.2666 | -0.8649 | -0.83 | 0.4053 |
| DC vs ME | 0 | 0 | -0.3308 | NA | -0.3308 | NA | NA | NA |
| DC vs SE | 0 | 0 | 0.2565 | NA | 0.2565 | NA | NA | NA |
| DC vs SG | 0 | 0 | -0.2958 | NA | -0.2958 | NA | NA | NA |
| ME vs SE | 0 | 0 | 0.5873 | NA | 0.5873 | NA | NA | NA |
| ME vs SG | 0 | 0 | 0.035 | NA | 0.035 | NA | NA | NA |
| SE vs SG | 0 | 0 | -0.5523 | NA | -0.5523 | NA | NA | NA |

**Table 5.3.2 Details of SIDE splitting results (Inhibition reaction time)**

| **comparison** | **k** | **prop** | **nma** | **direct** | **indir.** | **Diff** | **z** | **p-value** |
| --- | --- | --- | --- | --- | --- | --- | --- | --- |
| AE vs CON | 6 | 1 | 0.0125 | 0.0129 | NA | NA | NA | NA |
| AE vs DC | 0 | 0 | -0.0314 | NA | -0.0314 | NA | NA | NA |
| AE vs ME | 0 | 0 | -0.0185 | NA | -0.0185 | NA | NA | NA |
| AE vs SE | 0 | 0 | 0.0593 | NA | 0.0593 | NA | NA | NA |
| AE vs SG | 4 | 0.93 | 0.0265 | 0.0663 | -0.4647 | 0.531 | 0.62 | 0.5333 |
| DC vs CON | 3 | 1 | 0.0439 | 0.0439 | NA | NA | NA | NA |
| ME vs CON | 7 | 1 | 0.031 | 0.031 | NA | NA | NA | NA |
| SE vs CON | 5 | 1 | -0.0468 | -0.0468 | NA | NA | NA | NA |
| SG vs CON | 4 | 0.93 | -0.0141 | 0.0254 | -0.5183 | 0.5437 | 0.63 | 0.5281 |
| DC vs ME | 0 | 0 | 0.0129 | NA | 0.0129 | NA | NA | NA |
| DC vs SE | 0 | 0 | 0.0908 | NA | 0.0908 | NA | NA | NA |
| DC vs SG | 0 | 0 | 0.058 | NA | 0.058 | NA | NA | NA |
| ME vs SE | 0 | 0 | 0.0778 | NA | 0.0778 | NA | NA | NA |
| ME vs SG | 0 | 0 | 0.045 | NA | 0.045 | NA | NA | NA |
| SE vs SG | 0 | 0 | -0.0328 | NA | -0.0328 | NA | NA | NA |

NA not available, k Number of studies providing direct evidence, prop Direct evidence proportion, nma Estimated treatment effect in network meta-analysis, direct Estimated treatment effect derived from direct evidence, indir. Estimated treatment effect derived from indirect evidence, Diff direct versus indirect, z z-value of test for disagreement (direct versus indirect), p p-value of test for disagreement (direct versus indirect).

# Supplementary 6: Publication bias


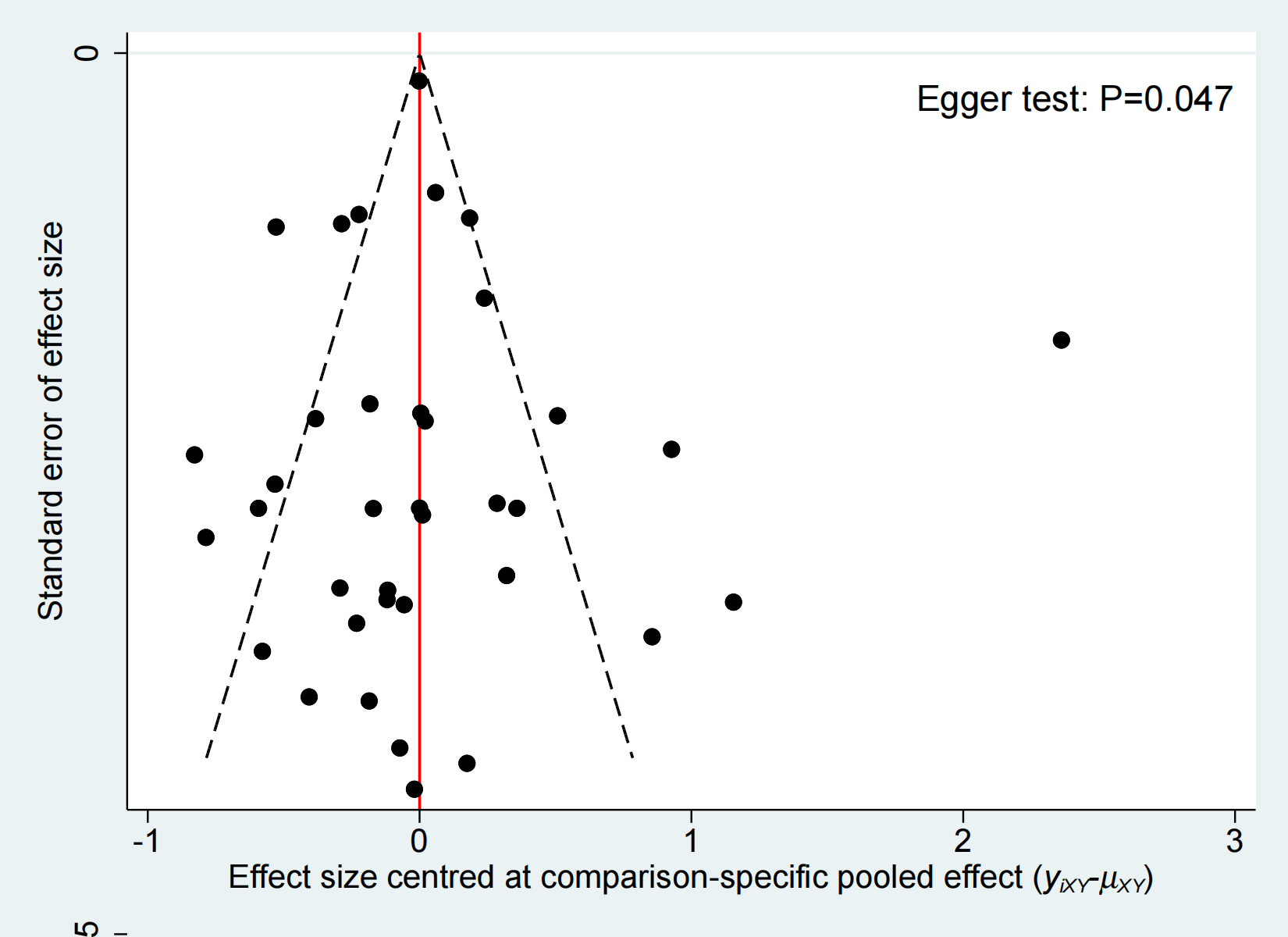


Figure 6.1 The funnel plot of inhibition accuracy. The result of Egger test showed the p=0.047.


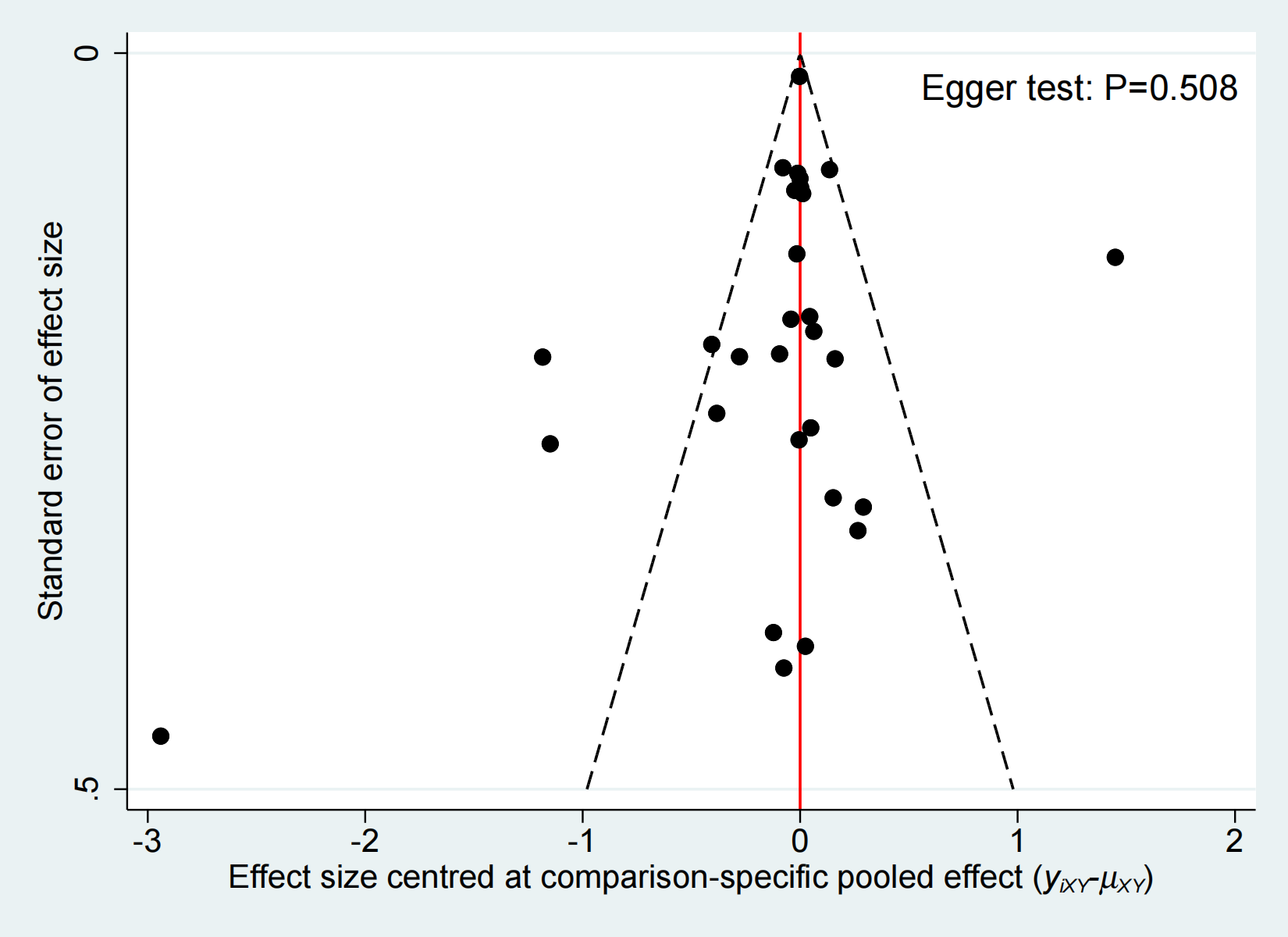


Figure 6.2 The funnel plot of inhibition reaction time. The result of Egger test showed the p=0.508.

# Supplementary 7: Forest plot


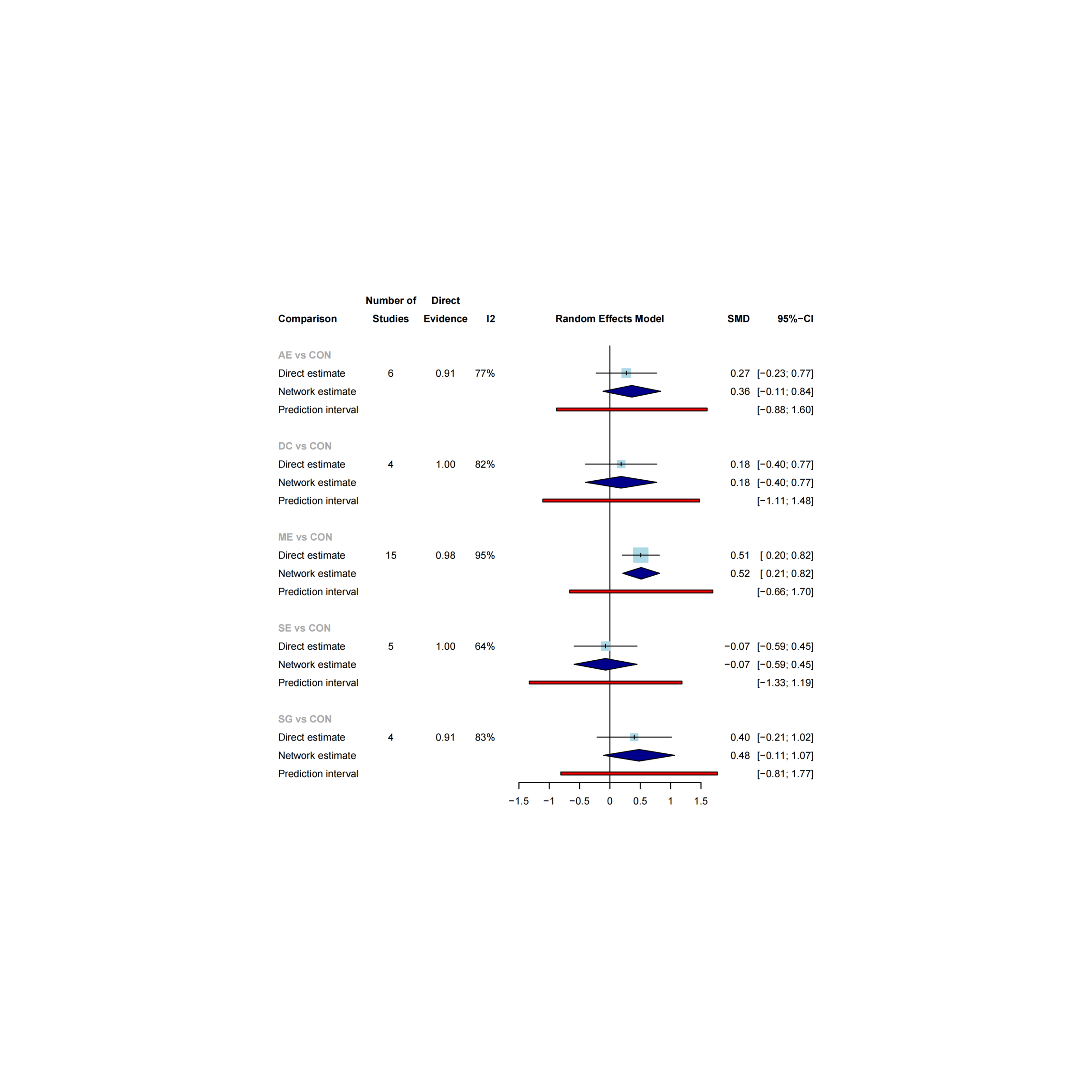


Figure 7.1 Forest plot of the effect of PA on inhibition accuracy. SMD standard mean difference, CI Credible Interval.


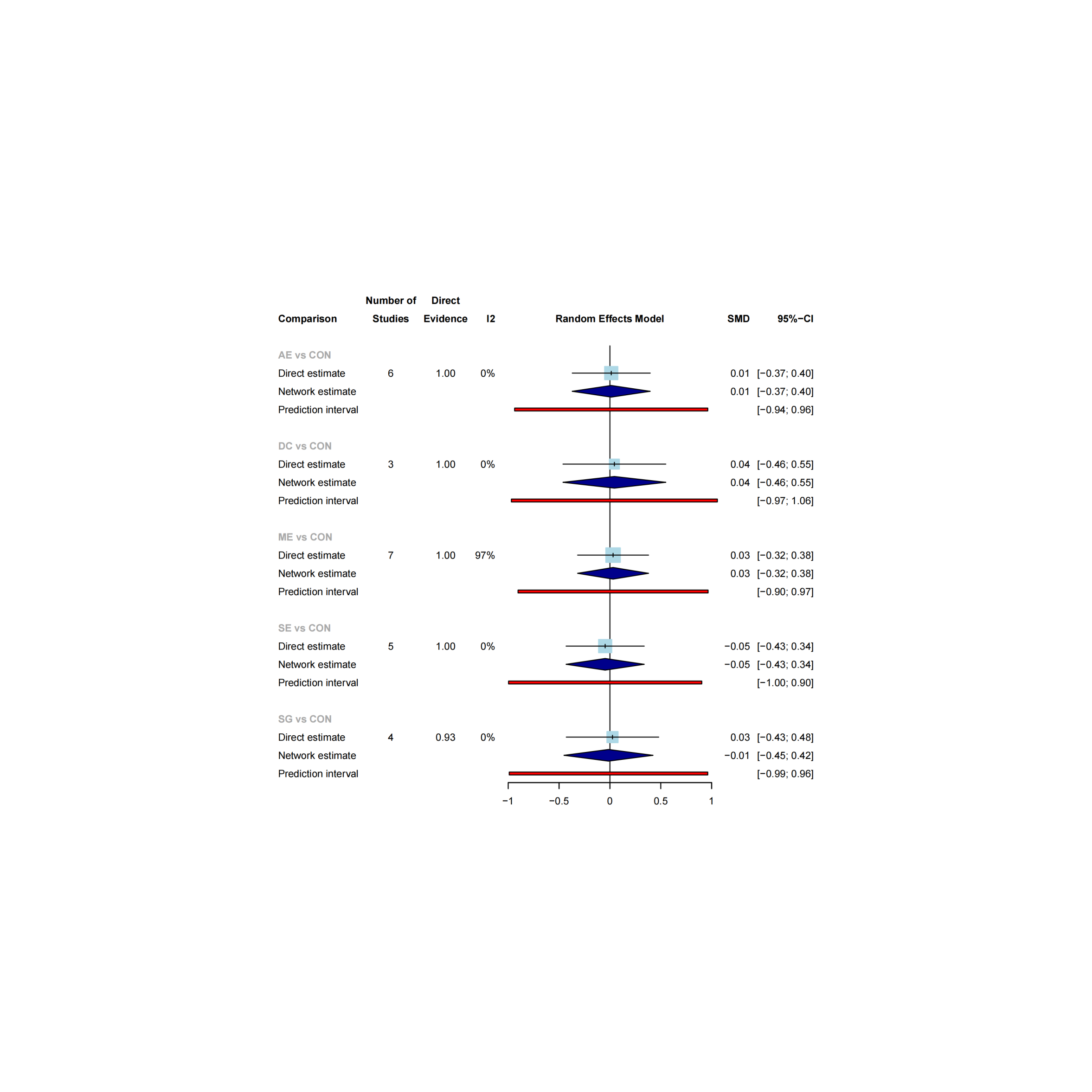


Figure 7.2 Forest plot of the effect of PA on inhibition reaction time. SMD standard mean difference,CI Credible Interval.

#

# Supplementary 8: Assessment of Model Consistency

We analyzed the data with the consistency model and the unrelated mean effect model, and compared the differences in the deviation, the number of estimated parameters in the network, and the Deviance Informative Criterion (DIC) indicators of the two models. If these are similar, it means that our research has good consistency^1^. Comparison of these parameters indicated good consistency across models (Table 11.1-6).

**Table 8.1. Consistent and UME models fit comparison (Inhibition accuracy)**

| **Model** | **pD** | **Residual deviance** | **Deviance** | **DIC** | **SD** |
| --- | --- | --- | --- | --- | --- |
| Consistent | 64.3 | 64.906 | 88.492 | 152.4 | 0.859 |
| UME | 61.3 | 63.757 | 87.343 | 148.1 | 0.701 |

pD: Number of estimated parameters; DIC: Deviance Informative Criterion; SD: Standard Deviation; UME: Unrelated Mean Effects. Scientific literature indicated that the main indicator to assess the model fit is the DIC. As lower DIC, better fit.

**Table 8.2. Consistent and UME models fit comparison (Inhibition reaction time)**

| **Model** | **pD** | **Residual deviance** | **Deviance** | **DIC** | **SD** |
| --- | --- | --- | --- | --- | --- |
| Consistent | 45.2 | 45.328 | 291.413 | 336.1 | 0.519 |
| UME | 44.1 | 46.750 | 292.835 | 336.4 | 0.742 |

pD: Number of estimated parameters; DIC: Deviance Informative Criterion; SD: Standard Deviation; UME: Unrelated Mean Effects. Scientific literature indicated that the main indicator to assess the model fit is the DIC. As lower DIC, better fit.

**Reference**

1. Wheeler DC, Hickson DA, Waller LA. Assessing Local Model Adequacy in Bayesian Hierarchical Models Using the Partitioned Deviance Information Criterion. Comput Stat Data Anal. 2010;54(6):1657-1671.

# Supplementary File 9: Node-splitting analysis of inconsistency

We assessed inconsistency via MBNMA node-splitting approach. This method splits and compares contributions for a particular intervention contrast into direct and indirect evidence^1^. Similar effects denote good consistency. Table 10.1 below present the results for node-splitting analysis of inconsistencyin this meta-analysis. The results showed that there was no inconsistency in our study (P>0.05).

**Table 9.1 Node-splitting analysis of inconsistency (Inhibition accuracy)**

| **Comparison** | **p-value** | **Median** | **2.50%** | **97.50%** |
| --- | --- | --- | --- | --- |
| **SG_1200 vs Placebo_0** | **0.621** |  |  |  |
| -> direct |  | 0.129 | -1.246 | 1.558 |
| -> indirect |  | 0.648 | -0.228 | 1.533 |
| -> MBNMA |  | 0.507 | -0.272 | 1.297 |
| **SG_600 vs Placebo_0** | **0.495** |  |  |  |
| -> direct |  | -0.083 | -1.309 | 1.153 |
| -> indirect |  | 0.608 | -0.245 | 1.455 |
| -> MBNMA |  | 0.482 | -0.252 | 1.223 |
| **SG_300 vs Placebo_0** | **0.69** |  |  |  |
| -> direct |  | 0.256 | -1.123 | 1.568 |
| -> indirect |  | 0.449 | -0.279 | 1.186 |
| -> MBNMA |  | 0.438 | -0.227 | 1.121 |
| **SG_200 vs Placebo_0** | **0.186** |  |  |  |
| -> direct |  | 1.49 | 0.142 | 2.805 |
| -> indirect |  | 0.155 | -0.464 | 0.81 |
| -> MBNMA |  | 0.398 | -0.197 | 1.052 |
| **SE_300 vs Placebo_0** | **0.675** |  |  |  |
| -> direct |  | 0.245 | -1.135 | 1.649 |
| -> indirect |  | -0.179 | -1.139 | 0.706 |
| -> MBNMA |  | -0.084 | -0.879 | 0.672 |
| **SE_200 vs Placebo_0** | **0.737** |  |  |  |
| -> direct |  | 0.023 | -1.392 | 1.293 |
| -> indirect |  | -0.077 | -0.881 | 0.738 |
| -> MBNMA |  | -0.076 | -0.802 | 0.607 |
| **SE_100 vs Placebo_0** | **0.687** |  |  |  |
| -> direct |  | -0.218 | -1.019 | 0.601 |
| -> indirect |  | 0.092 | -0.67 | 0.863 |
| -> MBNMA |  | -0.059 | -0.627 | 0.479 |
| **ME_1500 vs Placebo_0** | **0.471** |  |  |  |
| -> direct |  | 0.938 | -0.108 | 1.959 |
| -> indirect |  | 0.539 | 0.112 | 0.967 |
| -> MBNMA |  | 0.592 | 0.182 | 1.004 |
| **ME_1200 vs Placebo_0** | **0.328** |  |  |  |
| -> direct |  | -0.039 | -1.438 | 1.271 |
| -> indirect |  | 0.644 | 0.234 | 1.057 |
| -> MBNMA |  | 0.583 | 0.178 | 0.987 |
| **ME_1100 vs Placebo_0** | **0.02** |  |  |  |
| -> direct |  | 1.809 | 0.97 | 2.588 |
| -> indirect |  | 0.34 | -0.008 | 0.702 |
| -> MBNMA |  | 0.579 | 0.177 | 0.98 |
| **ME_800 vs Placebo_0** | **0.33** |  |  |  |
| -> direct |  | 0.075 | -0.863 | 0.995 |
| -> indirect |  | 0.672 | 0.256 | 1.052 |
| -> MBNMA |  | 0.564 | 0.172 | 0.949 |
| **ME_700 vs Placebo_0** | **0.435** |  |  |  |
| -> direct |  | 0.435 | -1.055 | 1.888 |
| -> indirect |  | 0.565 | 0.195 | 0.956 |
| -> MBNMA |  | 0.556 | 0.169 | 0.934 |
| **ME_600 vs Placebo_0** | **0.387** |  |  |  |
| -> direct |  | 0.214 | -1.269 | 1.711 |
| -> indirect |  | 0.567 | 0.196 | 0.96 |
| -> MBNMA |  | 0.546 | 0.166 | 0.917 |
| **ME_500 vs Placebo_0** | **0.313** |  |  |  |
| -> direct |  | -0.083 | -1.37 | 1.224 |
| -> indirect |  | 0.567 | 0.197 | 0.939 |
| -> MBNMA |  | 0.533 | 0.163 | 0.891 |
| **ME_300 vs Placebo_0** | **0.415** |  |  |  |
| -> direct |  | 0.531 | -0.872 | 1.918 |
| -> indirect |  | 0.492 | 0.121 | 0.814 |
| -> MBNMA |  | 0.485 | 0.148 | 0.822 |
| **ME_200 vs Placebo_0** | **0.486** |  |  |  |
| -> direct |  | 0.322 | -0.738 | 1.369 |
| -> indirect |  | 0.448 | 0.133 | 0.813 |
| -> MBNMA |  | 0.438 | 0.131 | 0.75 |
| **ME_100 vs Placebo_0** | **0.297** |  |  |  |
| -> direct |  | -0.092 | -1.599 | 1.452 |
| -> indirect |  | 0.352 | 0.115 | 0.717 |
| -> MBNMA |  | 0.335 | 0.096 | 0.658 |
| **DC_300 vs Placebo_0** | **0.62** |  |  |  |
| -> direct |  | -0.143 | -1.541 | 1.191 |
| -> indirect |  | 0.397 | -0.589 | 1.397 |
| -> MBNMA |  | 0.203 | -0.587 | 1.01 |
| **DC_200 vs Placebo_0** | **0.71** |  |  |  |
| -> direct |  | 0.378 | -0.623 | 1.43 |
| -> indirect |  | 0.005 | -0.967 | 0.97 |
| -> MBNMA |  | 0.184 | -0.536 | 0.904 |
| **DC_100 vs Placebo_0** | **0.663** |  |  |  |
| -> direct |  | 0.234 | -1.104 | 1.55 |
| -> indirect |  | 0.118 | -0.532 | 0.811 |
| -> MBNMA |  | 0.142 | -0.418 | 0.723 |
| **AE_1200 vs Placebo_0** | **0.643** |  |  |  |
| -> direct |  | 0.367 | -1.084 | 1.9 |
| -> indirect |  | 0.404 | -0.274 | 1.105 |
| -> MBNMA |  | 0.394 | -0.195 | 1.034 |
| **AE_900 vs Placebo_0** | **0.634** |  |  |  |
| -> direct |  | 0.392 | -1.034 | 1.884 |
| -> indirect |  | 0.387 | -0.286 | 1.06 |
| -> MBNMA |  | 0.386 | -0.193 | 1.006 |
| **AE_600 vs Placebo_0** | **0.342** |  |  |  |
| -> direct |  | 1.105 | -0.253 | 2.457 |
| -> indirect |  | 0.225 | -0.393 | 0.879 |
| -> MBNMA |  | 0.372 | -0.186 | 0.963 |
| **AE_500 vs Placebo_0** | **0.554** |  |  |  |
| -> direct |  | 0.099 | -1.498 | 1.79 |
| -> indirect |  | 0.396 | -0.208 | 0.992 |
| -> MBNMA |  | 0.363 | -0.184 | 0.937 |
| **AE_300 vs Placebo_0** | **0.346** |  |  |  |
| -> direct |  | -0.153 | -1.075 | 0.749 |
| -> indirect |  | 0.548 | -0.059 | 1.164 |
| -> MBNMA |  | 0.331 | -0.168 | 0.858 |

The number after the PA type represents the weekly dose (MET-min). AE aerobic exercise, DC dance, ME mixed exercise, SE snack exercise, SG sports game.

**Table 9.2 Node-splitting analysis of inconsistency (Inhibition reaction time)**

| **Comparison** | **p-value** | **Median** | **2.50%** | **97.50%** |
| --- | --- | --- | --- | --- |
| **SG_600 vs Placebo_0** | **0.734** |  |  |  |
| -> direct |  | 0.203 | -1.23 | 1.604 |
| -> indirect |  | -0.052 | -0.989 | 0.842 |
| -> MBNMA |  | -0.028 | -0.833 | 0.743 |
| **SG_500 vs Placebo_0** | **0.834** |  |  |  |
| -> direct |  | 0.014 | -1.327 | 1.387 |
| -> indirect |  | -0.028 | -0.949 | 0.856 |
| -> MBNMA |  | -0.027 | -0.813 | 0.725 |
| **SG_400 vs Placebo_0** | **0.766** |  |  |  |
| -> direct |  | -0.036 | -1.403 | 1.392 |
| -> indirect |  | -0.026 | -0.895 | 0.802 |
| -> MBNMA |  | -0.026 | -0.779 | 0.7 |
| **SG_300 vs Placebo_0** | **0.682** |  |  |  |
| -> direct |  | -0.077 | -1.569 | 1.368 |
| -> indirect |  | 0.005 | -0.775 | 0.783 |
| -> MBNMA |  | -0.024 | -0.746 | 0.656 |
| **SE_300 vs Placebo_0** | **0.797** |  |  |  |
| -> direct |  | -0.016 | -1.697 | 1.624 |
| -> indirect |  | -0.062 | -1.098 | 1.021 |
| -> MBNMA |  | -0.051 | -0.949 | 0.815 |
| **SE_200 vs Placebo_0** | **0.742** |  |  |  |
| -> direct |  | 0.061 | -1.547 | 1.635 |
| -> indirect |  | -0.081 | -1.028 | 0.855 |
| -> MBNMA |  | -0.046 | -0.842 | 0.72 |
| **SE_100 vs Placebo_0** | **0.891** |  |  |  |
| -> direct |  | -0.103 | -0.985 | 0.814 |
| -> indirect |  | 0.011 | -0.883 | 0.952 |
| -> MBNMA |  | -0.035 | -0.686 | 0.559 |
| **ME_1500 vs Placebo_0** | **0.413** |  |  |  |
| -> direct |  | -0.717 | -2.299 | 0.857 |
| -> indirect |  | 0.076 | -0.604 | 0.755 |
| -> MBNMA |  | -0.076 | -0.724 | 0.586 |
| **ME_1200 vs Placebo_0** | **0.597** |  |  |  |
| -> direct |  | 0.095 | -1.582 | 1.736 |
| -> indirect |  | -0.101 | -0.809 | 0.615 |
| -> MBNMA |  | -0.074 | -0.716 | 0.576 |
| **ME_1100 vs Placebo_0** | **0.008** |  |  |  |
| -> direct |  | 1.214 | 0.434 | 1.964 |
| -> indirect |  | -0.654 | -1.206 | -0.137 |
| -> MBNMA |  | -0.074 | -0.712 | 0.57 |
| **ME_300 vs Placebo_0** | **0.003** |  |  |  |
| -> direct |  | -2.525 | -3.793 | -1.281 |
| -> indirect |  | 0.235 | -0.17 | 0.635 |
| -> MBNMA |  | -0.063 | -0.627 | 0.46 |
| **ME_200 vs Placebo_0** | **0.37** |  |  |  |
| -> direct |  | -0.723 | -2.324 | 0.909 |
| -> indirect |  | 0.019 | -0.517 | 0.524 |
| -> MBNMA |  | -0.058 | -0.587 | 0.417 |
| **DC_300 vs Placebo_0** | **0.906** |  |  |  |
| -> direct |  | -0.007 | -1.517 | 1.522 |
| -> indirect |  | 0.159 | -1.189 | 1.625 |
| -> MBNMA |  | 0.043 | -0.976 | 1.067 |
| **DC_200 vs Placebo_0** | **0.773** |  |  |  |
| -> direct |  | 0.238 | -1.386 | 1.941 |
| -> indirect |  | -0.016 | -1.173 | 1.135 |
| -> MBNMA |  | 0.039 | -0.87 | 0.957 |
| **DC_100 vs Placebo_0** | **0.713** |  |  |  |
| -> direct |  | -0.063 | -1.632 | 1.535 |
| -> indirect |  | 0.08 | -0.78 | 0.969 |
| -> MBNMA |  | 0.03 | -0.692 | 0.754 |
| **AE_1200 vs Placebo_0** | **0.744** |  |  |  |
| -> direct |  | -0.202 | -1.418 | 0.925 |
| -> indirect |  | 0.107 | -0.781 | 0.994 |
| -> MBNMA |  | -0.013 | -0.686 | 0.668 |
| **AE_500 vs Placebo_0** | **0.744** |  |  |  |
| -> direct |  | 0.099 | -0.704 | 0.901 |
| -> indirect |  | -0.179 | -1.212 | 0.909 |
| -> MBNMA |  | -0.012 | -0.632 | 0.601 |

The number after the PA type represents the weekly dose (MET-min). AE aerobic exercise, DC dance, ME mixed exercise, SE snack exercise, SG sports game.

**Reference**

1. van Valkenhoef G, Dias S, Ades AE, Welton NJ. Automated generation of node-splitting models for assessment of inconsistency in network meta-analysis. Res Synth Methods. 2016 Mar;7(1):80-93. doi: 10.1002/jrsm.1167. Epub 2015 Oct 13. PMID: 26461181; PMCID: PMC5057346.

# Supplementary File 10: Non-linear functions and models fit comparison

The different doses of PA were meta-analysed as independent and unrelated intervention (i.e., “split” NMA). This step is useful to determine which function fits the data better and should subsequently be used in a Model-Based Network Meta-Analysis (MBNMA)^1^.

**
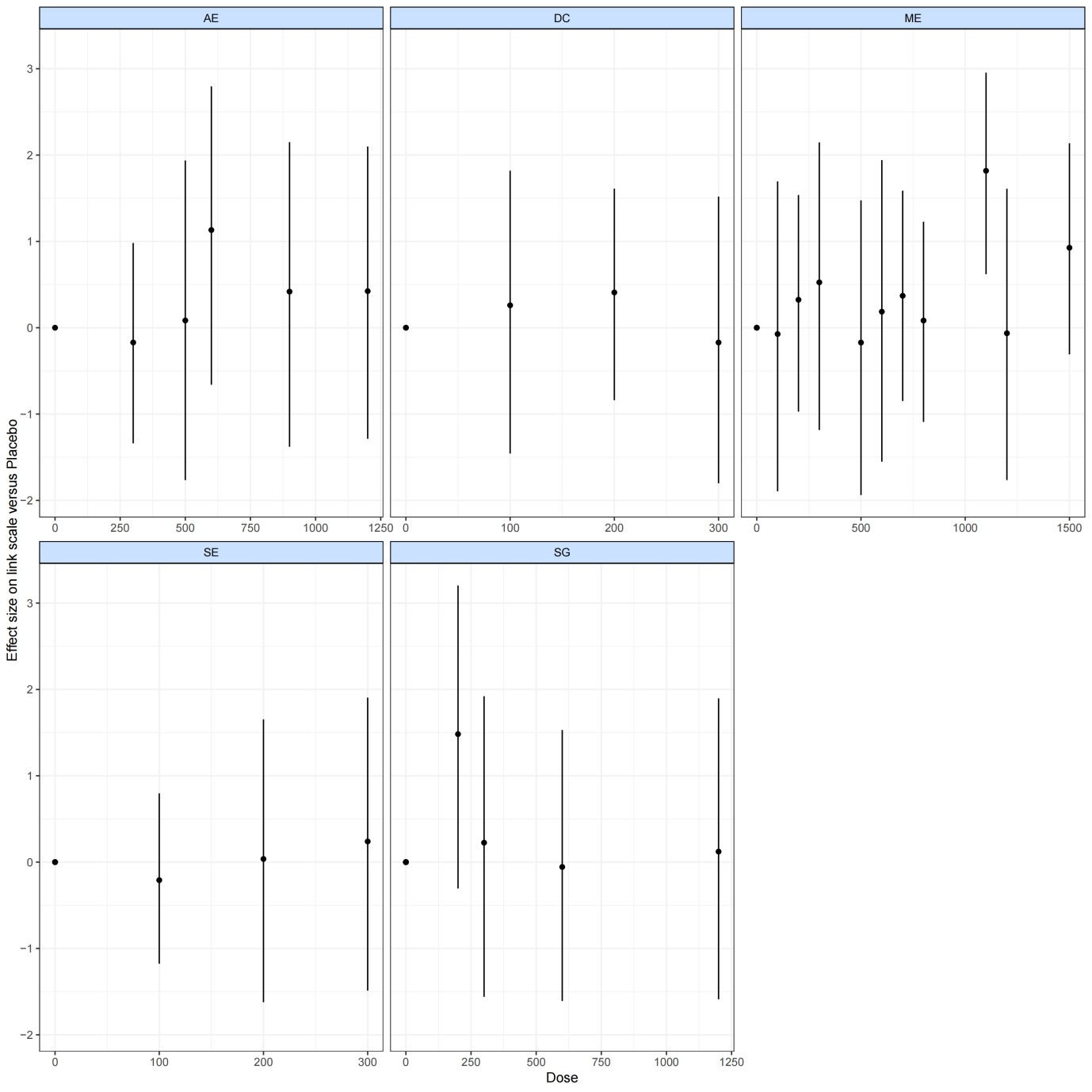
**

Figure 10.1 “Split” NMA of different PA intervention types on inhibition accuracy. AE aerobic exercise, CON control group, DC dance, ME mixed exercise, SE snack exercise, SG sports game.

**
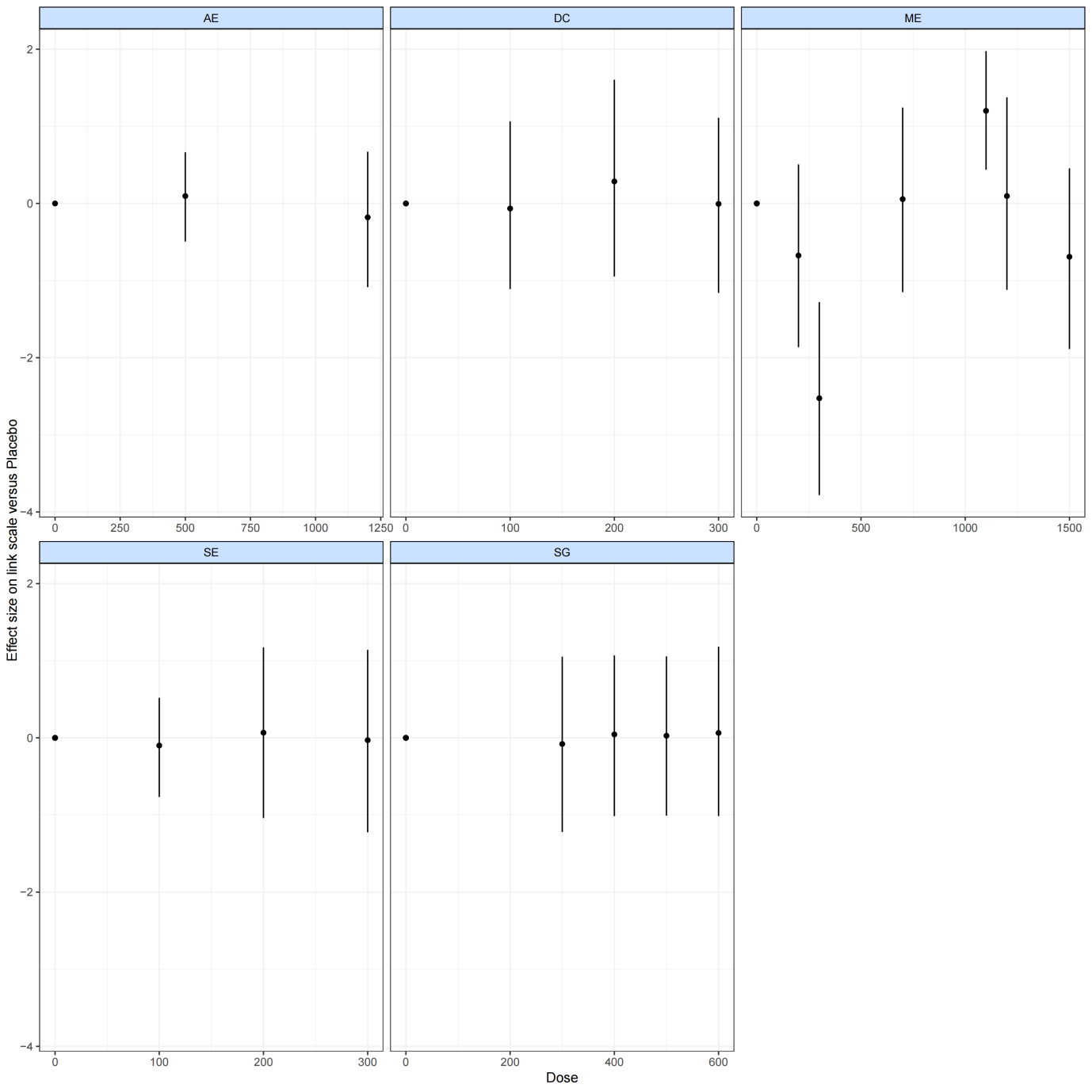
**

Figure 10.2 “Split” NMA of different PA intervention types on inhibition reaction time. AE aerobic exercise, CON control group, DC dance, ME mixed exercise, SE snack exercise, SG sports game.

**Table 10.1. Models fit comparison (Inhibition accuracy)**

| **Model** | **DIC** | **SD** | **Deviance** | **Residual deviance** | **pD** |
| --- | --- | --- | --- | --- | --- |
| **Emax**  **(common treatment effects)** | 621.5 | NA | 593.930 | 570.343 | 35.9 |
| **Emax**  **(random treatment effects)** | 147.6 | 0.658 | 87.174 | 63.588 | 61.1 |
| **Exponential**  **(common treatment effects)** | 610.6 | NA | 574.772 | 551.185 | 36.5 |
| **Exponential**  **(random treatment effects)** | 148.2 | 0.660 | 87.001 | 63.415 | 61.6 |
| **Restricted cubic spline**  **(common treatment effects; 3 knots)** | 569.1 | NA | 528.614 | 505.027 | 41.0 |
| **Restricted cubic spline**  **(random treatment effects; 3 knots)** | 147.1 | 0.648 | 87.111 | 63.213 | 60.7 |
| **Non-parametric monotonically up (common treatment effects)** | 458.3 | NA | 421.620 | 398.034 | 37.2 |
| **Non-parametric monotonically up (random treatment effects)** | 150.3 | 0.960 | 88.304 | 64.718 | 62.9 |

DIC = Deviance Information Criterion; SD = Between-study Standard Deviation; pD: Number of estimated parameters; NA = Not Applicable. The SD is presented as the main value and (95% Credible Intervals).

**Table 10.2. Models fit comparison (Inhibition reaction time)**

| **Model** | **DIC** | **SD** | **Deviance** | **Residual deviance** | **pD** |
| --- | --- | --- | --- | --- | --- |
| **Emax**  **(common treatment effects)** | 589.0 | NA | 563.557 | 317.472 | 26.2 |
| **Emax**  **(random treatment effects)** | 336.6 | 0.749 | 292.892 | 46.807 | 44.4 |
| **Exponential**  **(common treatment effects)** | 590.9 | NA | 564.319 | 318.234 | 26.0 |
| **Exponential**  **(random treatment effects)** | 335.8 | 0.752 | 292.541 | 46.456 | 44.2 |
| **Restricted cubic spline**  **(common treatment effects; 3 knots)** | 569.0 | NA | 538.489 | 292.404 | 31.1 |
| **Restricted cubic spline**  **(random treatment effects; 3 knots)** | 335.2 | 0.746 | 292.476 | 46.691 | 44.1 |
| **Non-parametric monotonically up (common treatment effects)** | 584.5 | NA | 560.157 | 314.071 | 27.3 |
| **Non-parametric monotonically up (random treatment effects)** | 336.9 | 1.538 | 292.177 | 46.091 | 45.1 |

DIC = Deviance Information Criterion; SD = Between-study Standard Deviation; pD: Number of estimated parameters; NA = Not Applicable. The SD is presented as the main value and (95% Credible Intervals).


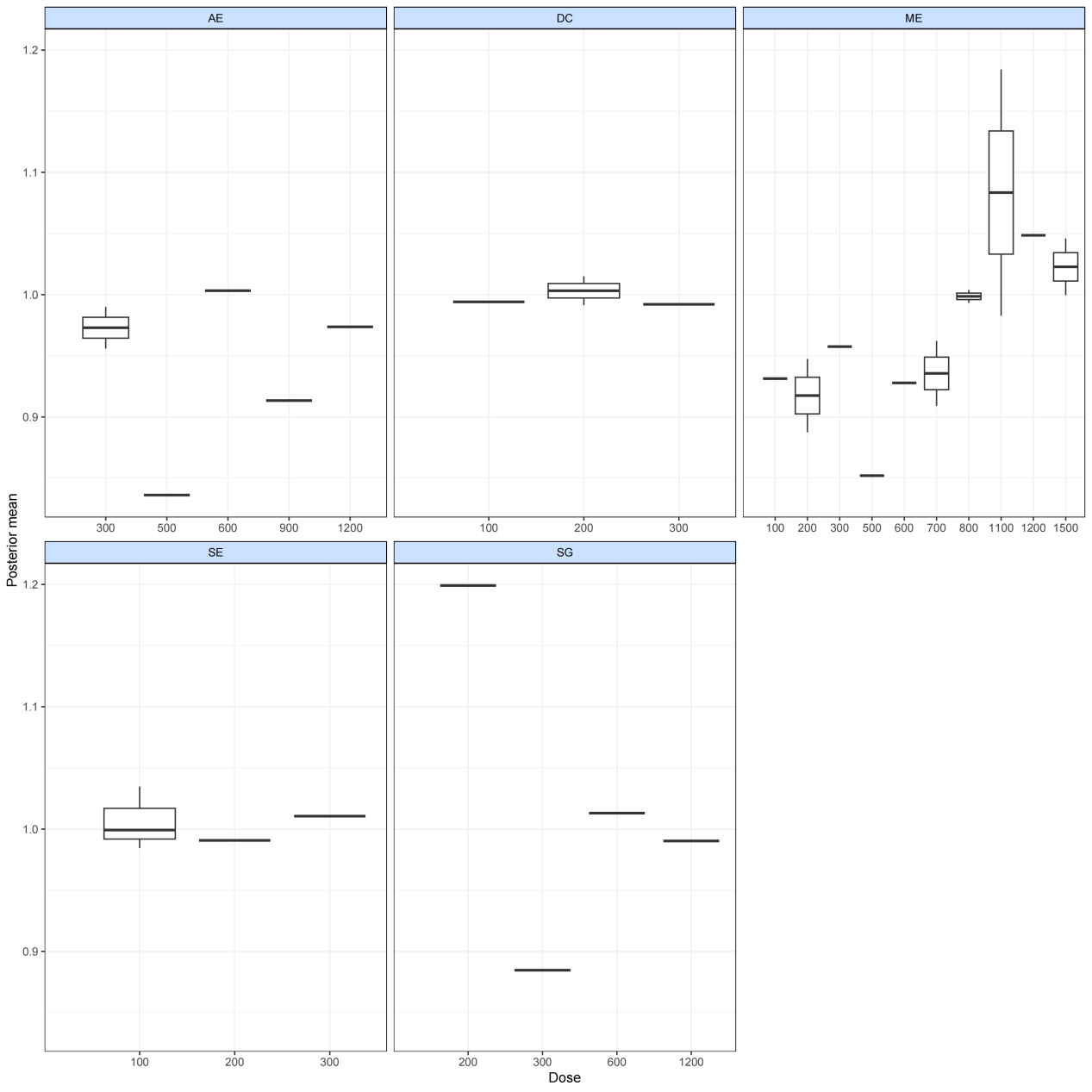


Figure 10.3. Deviance plots at intervention-level on inhibition accuracy. AE aerobic exercise, CON control group, DC dance, ME mixed exercise, SE snack exercise, SG sports game.


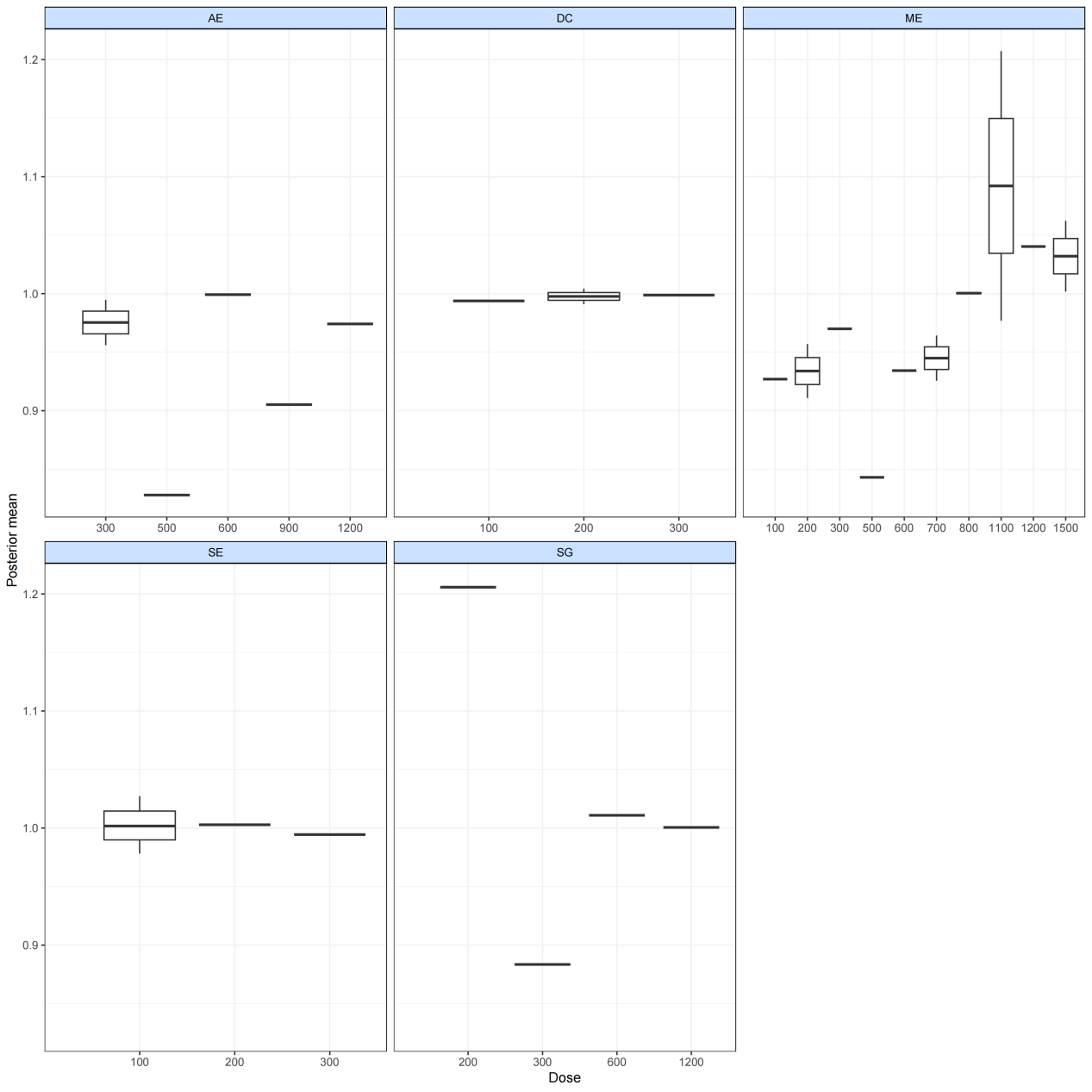


Figure 10.4. Deviance plots at intervention-level on inhibition reaction time. AE aerobic exercise, CON control group, DC dance, ME mixed exercise, SE snack exercise, SG sports game.

**Reference**

1. Pedder H. MBNMAdose: An R package for incorporating dose-response information into Network Meta-Analysis. Paper presented at: Evidence Synthesis and Meta-Analysis in R Conference 20212021.
2. Dias S, Sutton AJ, Ades AE, Welton NJ. Evidence synthesis for decision making 2: a generalized linear modeling framework for pairwise and network meta-analysis of randomized controlled trials. Medical Decision Making : an International Journal of the Society For Medical Decision Making. 2013;33(5):607-617.

# Supplementary File 11: Grading the evidence for outcome of the network meta-analysis using CINeMA


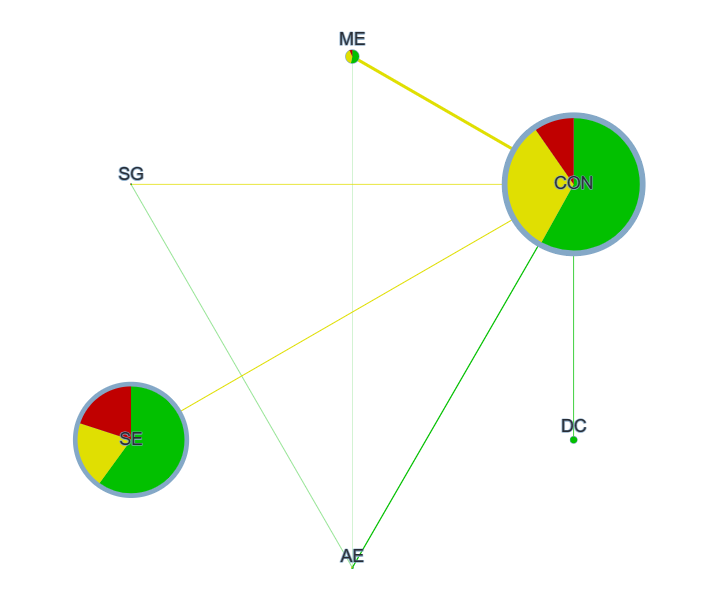


Figure 11.1 Network plot of study limitations of the included inhibition accuracy studies. Node size by equal size, node color by RoB. The colors in the circles indicate the percentage of low RoB studies (green), moderate RoB studies (yellow), high RoB studies (red) about each exercise type. Edge width by sample size. Edge color by average RoB. The colors of the lines indicate the summative RoB assessment of each comparison. Low RoB is green, moderate RoB is yellow, high RoB is red. AE Aerobic Exercise, CON Control group, DC Dance, ME Mixed Exercise, SE Snack Exercise, SG Sports Game.


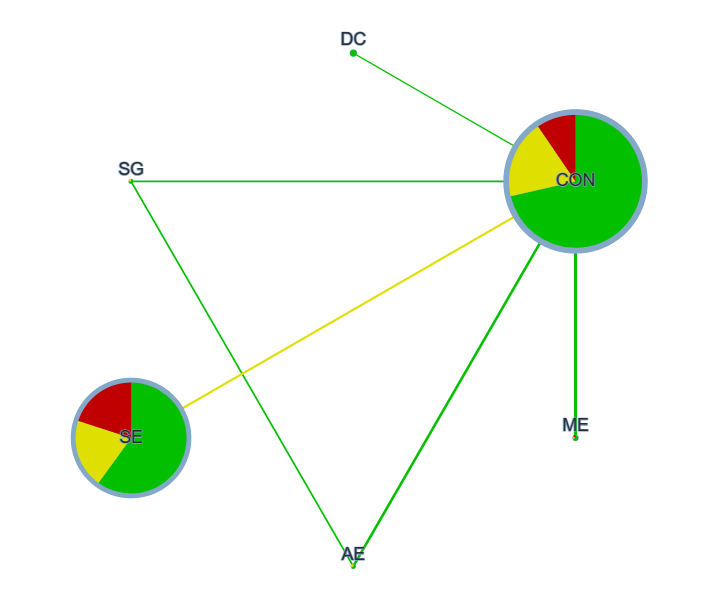


Figure 11.2 Network plot of study limitations of the included inhibition reaction time studies. Node size by equal size, node color by RoB. The colors in the circles indicate the percentage of low RoB studies (green), moderate RoB studies (yellow), high RoB studies (red) about each exercise type. Edge width by sample size. Edge color by average RoB. The colors of the lines indicate the summative RoB assessment of each comparison. Low RoB is green, moderate RoB is yellow, high RoB is red. AE Aerobic Exercise, CON Control group, DC Dance, ME Mixed Exercise, SE Snack Exercise, SG Sports Game.

##

Low RoB is green, moderate RoB is yellow, high RoB is red.


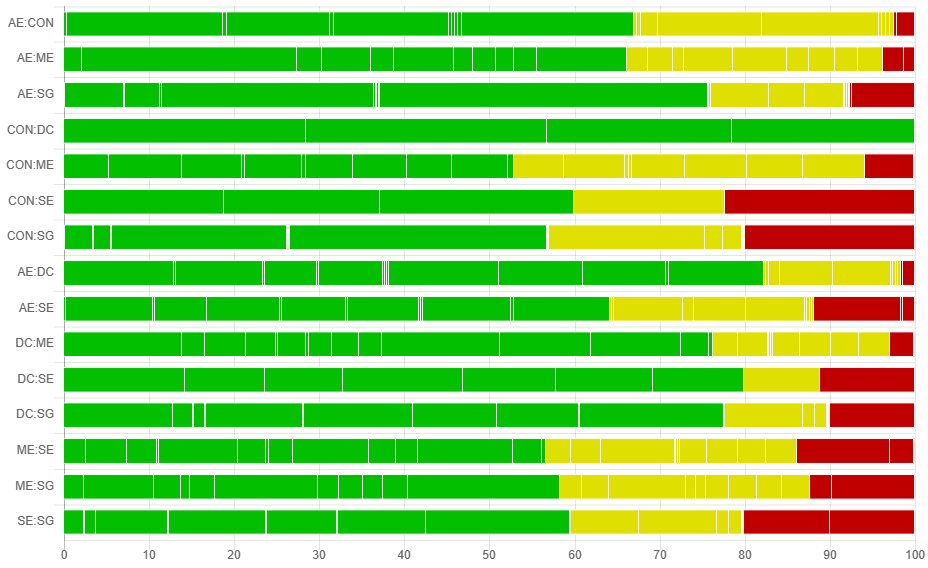


Figure 11.3 Plot of the percentage contribution of RoB comparisons to the estimation of inhibition accuracy across networks.


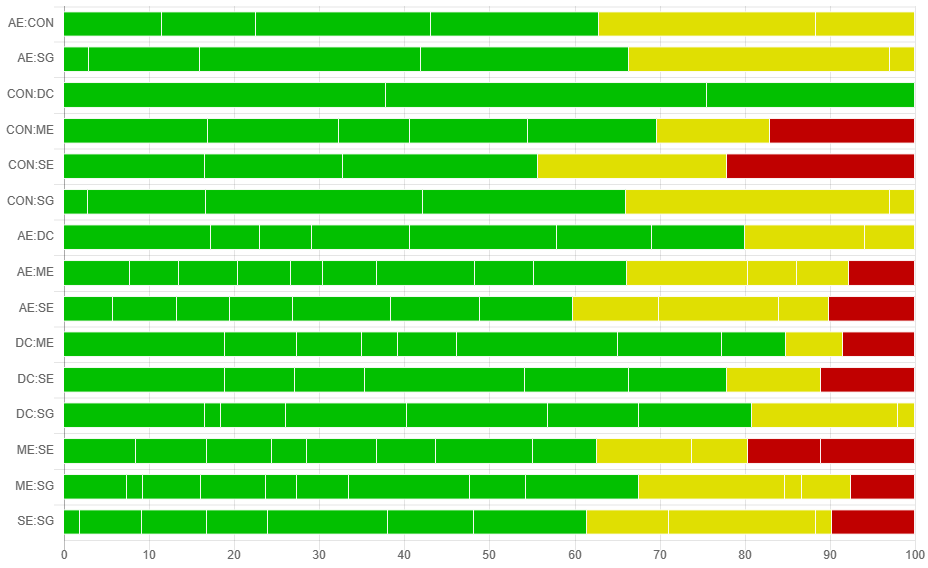
Figure 11.4 Plot of the percentage contribution of RoB comparisons to the estimation of inhibition reaction time across networks.

Table 11.1 CINeMA for the inhibition accuracy

| **Comparison** | **Number of studies** | **Within-study bias** | **Reporting bias** | **Indirectness** | **Imprecision** | **Heterogeneity** | **Incoherence** | **Confidence rating** |
| --- | --- | --- | --- | --- | --- | --- | --- | --- |
| AE:CON | 6 | No concerns | Low risk | No concerns | Major concerns | No concerns | No concerns | Low |
| AE:ME | 1 | No concerns | Low risk | No concerns | Major concerns | No concerns | No concerns | Low |
| AE:SG | 2 | No concerns | Low risk | No concerns | Major concerns | No concerns | No concerns | Low |
| CON:DC | 4 | No concerns | Low risk | No concerns | Major concerns | No concerns | No concerns | Low |
| CON:ME | 15 | Some concerns | Low risk | No concerns | No concerns | Major concerns | No concerns | Very low |
| CON:SE | 5 | Some concerns | Low risk | No concerns | Major concerns | No concerns | No concerns | Very low |
| CON:SG | 4 | Some concerns | Low risk | No concerns | Major concerns | No concerns | No concerns | Very low |
| AE:DC | 0 | No concerns | Low risk | No concerns | Major concerns | No concerns | No concerns | Low |
| AE:SE | 0 | No concerns | Low risk | No concerns | Major concerns | No concerns | No concerns | Low |
| DC:ME | 0 | No concerns | Low risk | No concerns | Major concerns | No concerns | No concerns | Low |
| DC:SE | 0 | No concerns | Low risk | No concerns | Major concerns | No concerns | No concerns | Low |
| DC:SG | 0 | No concerns | Low risk | No concerns | Major concerns | No concerns | No concerns | Low |
| ME:SE | 0 | Some concerns | Low risk | No concerns | Major concerns | No concerns | No concerns | Very low |
| ME:SG | 0 | Some concerns | Low risk | No concerns | Major concerns | No concerns | No concerns | Very low |
| SE:SG | 0 | Some concerns | Low risk | No concerns | Major concerns | No concerns | No concerns | Very low |

AE Aerobic Exercise, CON Control group, DC Dance, ME Mixed Exercise, SE Snack Exercise, SG Sports Game.

Table 11.2 CINeMA for the inhibition reaction time

| **Comparison** | **Number of studies** | **Within-study bias** | **Reporting bias** | **Indirectness** | **Imprecision** | **Heterogeneity** | **Incoherence** | **Confidence rating** |
| --- | --- | --- | --- | --- | --- | --- | --- | --- |
| AE:CON | 6 | No concerns | Low risk | No concerns | Major concerns | No concerns | No concerns | Low |
| AE:SG | 4 | No concerns | Low risk | No concerns | Major concerns | No concerns | No concerns | Low |
| CON:DC | 3 | No concerns | Low risk | No concerns | Major concerns | No concerns | No concerns | Low |
| CON:ME | 7 | No concerns | Low risk | No concerns | Major concerns | No concerns | No concerns | Low |
| CON:SE | 5 | Some concerns | Low risk | No concerns | Major concerns | No concerns | No concerns | Very low |
| CON:SG | 4 | No concerns | Low risk | No concerns | Major concerns | No concerns | No concerns | Low |
| AE:DC | 0 | No concerns | Low risk | No concerns | Major concerns | No concerns | No concerns | Low |
| AE:ME | 0 | No concerns | Low risk | No concerns | Major concerns | No concerns | No concerns | Low |
| AE:SE | 0 | Some concerns | Low risk | No concerns | Major concerns | No concerns | No concerns | Very low |
| DC:ME | 0 | No concerns | Low risk | No concerns | Major concerns | No concerns | No concerns | Low |
| DC:SE | 0 | No concerns | Low risk | No concerns | Major concerns | No concerns | No concerns | Low |
| DC:SG | 0 | No concerns | Low risk | No concerns | Major concerns | No concerns | No concerns | Low |
| ME:SE | 0 | Some concerns | Low risk | No concerns | Major concerns | No concerns | No concerns | Very low |
| ME:SG | 0 | No concerns | Low risk | No concerns | Major concerns | No concerns | No concerns | Low |
| SE:SG | 0 | No concerns | Low risk | No concerns | Major concerns | No concerns | No concerns | Low |

AE Aerobic Exercise, CON Control group, DC Dance, ME Mixed Exercise, SE Snack Exercise, SG Sports Game.

# Supplementary File 12. Sensitivity analysis by inhibitory control task type

| **Task Type** | **Number of Studies** | **Effect of Mixed Exercise**  **(SMD, 95% CI)** | **Consistency of Ranking vs. Main Analysis** | **Notes** |
| --- | --- | --- | --- | --- |
| **Flanker Task** | 12 | **0.48 (0.18–0.77)** | **Consistent** | ME remained the top-ranked intervention for accuracy. |
| **Go/No-Go Task** | 10 | **0.51 (0.16–0.82)** | **Consistent** | Effects stable; SE improved reaction time but not accuracy. |
| **Stroop Task** | 9 | **0.46 (0.10–0.79)** | **Consistent** | ME retained highest SUCRA; task complexity did not alter rankings. |
